# Supplementary material for: Improved CRISPR/Cas9 off-target prediction with DNABERT and epigenetic features
Source: PLoS One. 2025 Nov 12;20(11):e0335863. doi: 10.1371/journal.pone.0335863 (PMC12611124; doi:10.1371/journal.pone.0335863)
Supplement: S1 File — (PDF) [file pone.0335863.s001.pdf]

Supplementary Figures 1: Prediction Performance

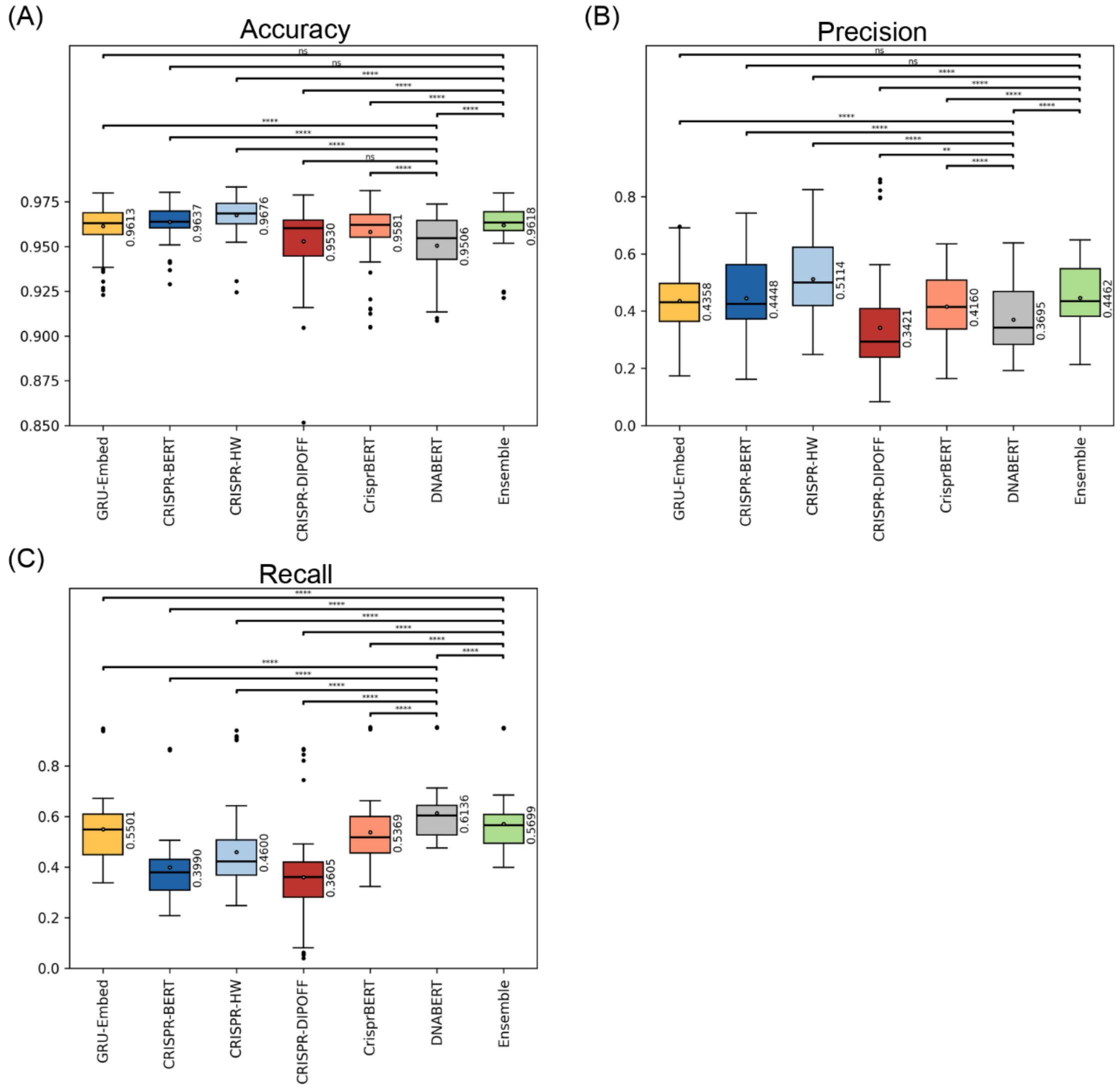

S1 Fig1. Performance comparison of all models on the Lazzarotto *et al*. (2020) CHANGE-seq dataset (1).

Boxplots show the distribution of (A) Accuracy, (B) Precision, and (C) Recall scores from the cross-validation experiments. The central line in each box indicates the median, the box represents the interquartile range (IQR), and the whiskers extend to 1.5 times the IQR. Dots beyond the whiskers are outliers. Statistical significance between model pairs was determined using the two-sided Wilcoxon signed-rank test with Benjamini-Hochberg correction. Significance levels are denoted as follows: ns: $p > 0.05$, *: $p \leq 0.05$, **: $p \leq 0.01$, ***: $p \leq 0.001$, ****: $p \leq 0.0001$.

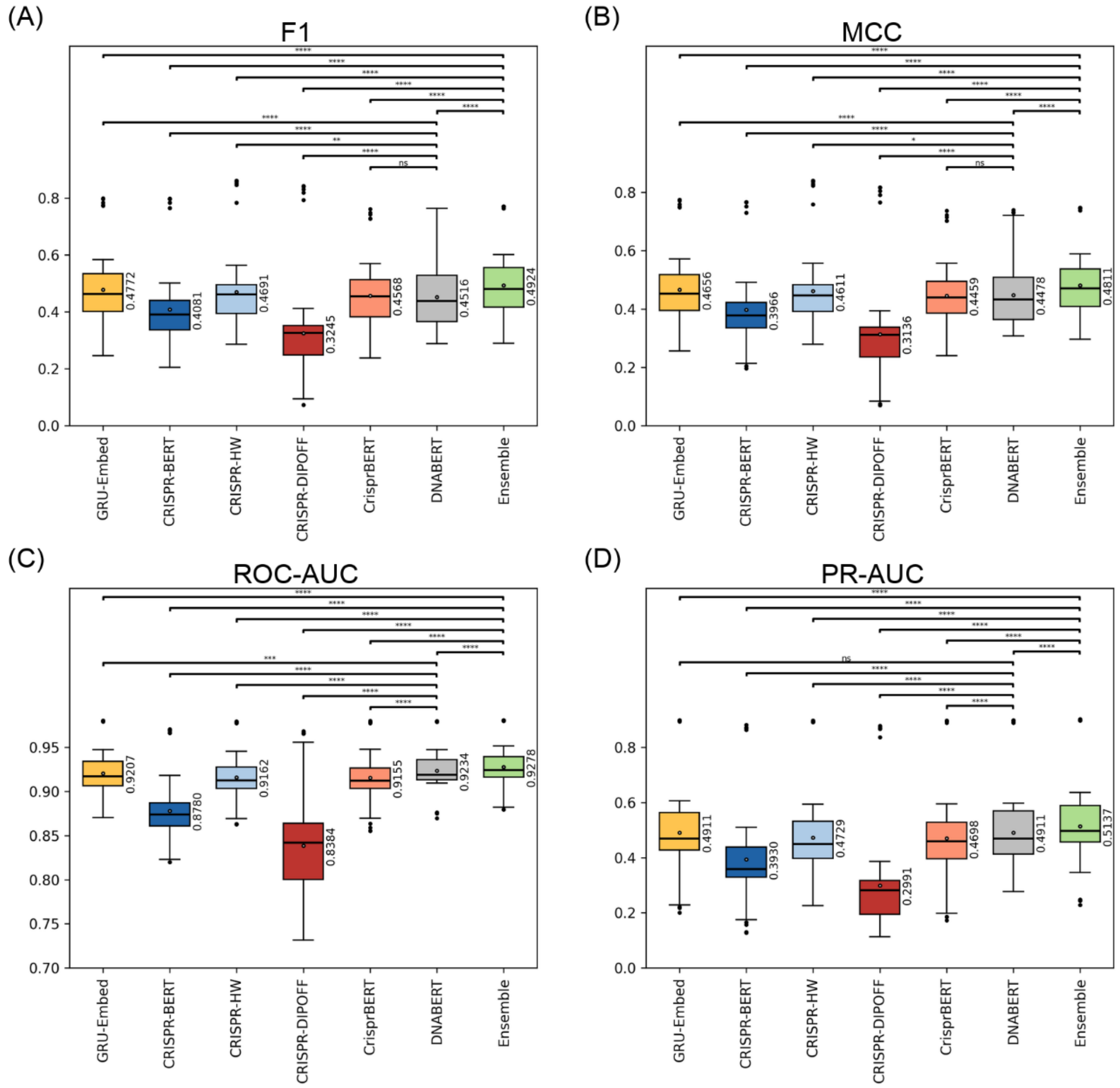

S1 Fig2. Performance comparison of all models on the Lazzarotto *et al*. (2020) CHANGE-seq dataset (2).

Boxplots show the distribution of (A) F1-score, (B) MCC, (C) ROC-AUC, and (D) PR-AUC scores from the cross-validation experiments. The central line in each box indicates the median, the box represents the interquartile range (IQR), and the whiskers extend to 1.5 times the IQR. Dots beyond the whiskers are outliers. Statistical significance between model pairs was determined using the two-sided Wilcoxon signed-rank test with Benjamini-Hochberg correction. Significance levels are denoted as follows: ns: $p > 0.05$, *: $p \leq 0.05$, **: $p \leq 0.01$, ***: $p \leq 0.001$, ****: $p \leq 0.0001$.

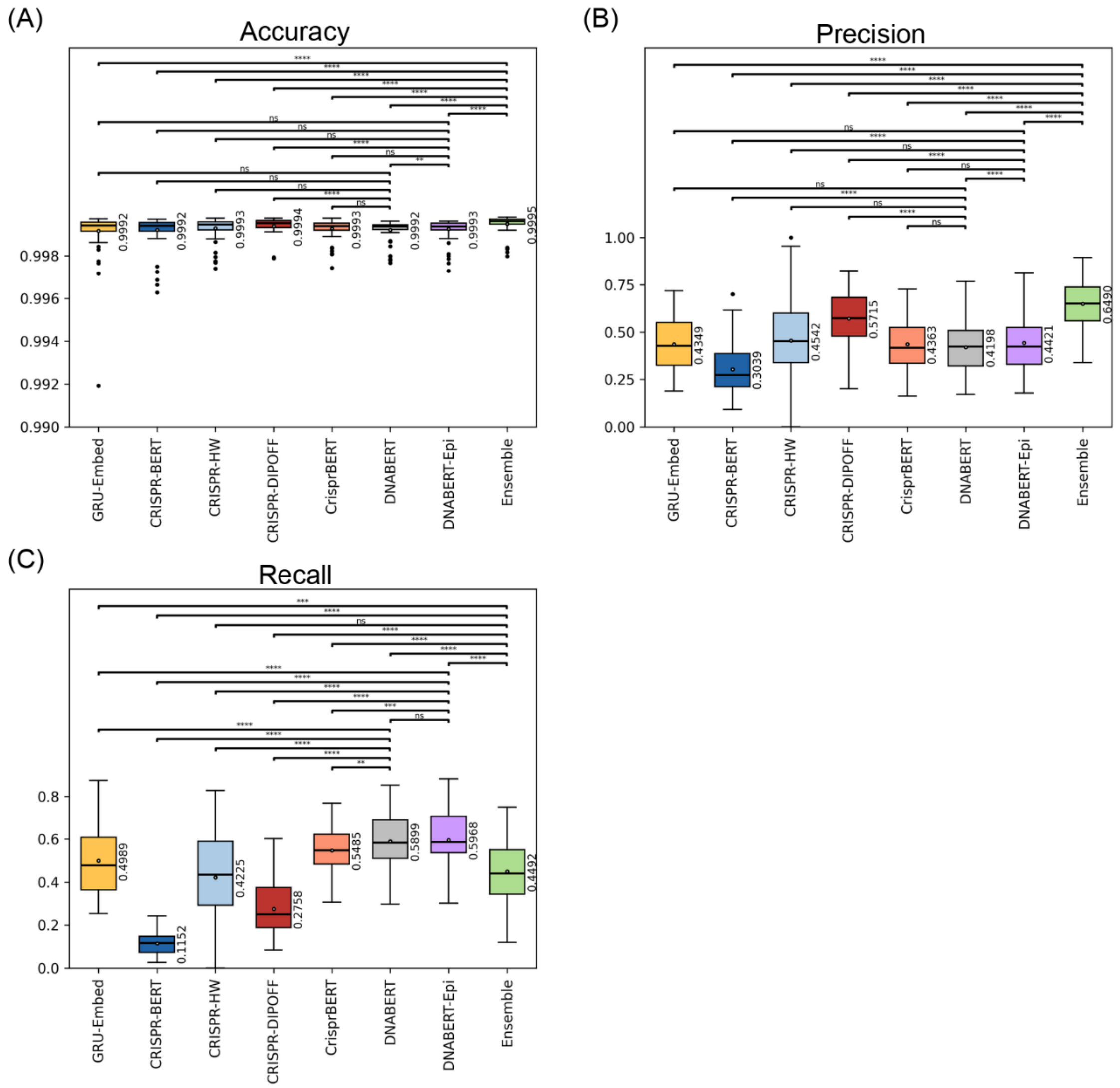

S1 Fig3. Performance comparison of all models on the Lazzarotto *et al*. (2020) GUIDE-seq dataset for additional metrics.

Boxplots show the distribution of (A) Accuracy, (B) Precision, and (C) Recall scores from the cross-validation experiments. The central line in each box indicates the median, the box represents the interquartile range (IQR), and the whiskers extend to 1.5 times the IQR. Dots beyond the whiskers are outliers. Statistical significance between model pairs was determined using the two-sided Wilcoxon signed-rank test with Benjamini-Hochberg correction. Significance levels are denoted as follows: ns: $p > 0.05$, *: $p \leq 0.05$, **: $p \leq 0.01$, ***: $p \leq 0.001$, ****: $p \leq 0.0001$.

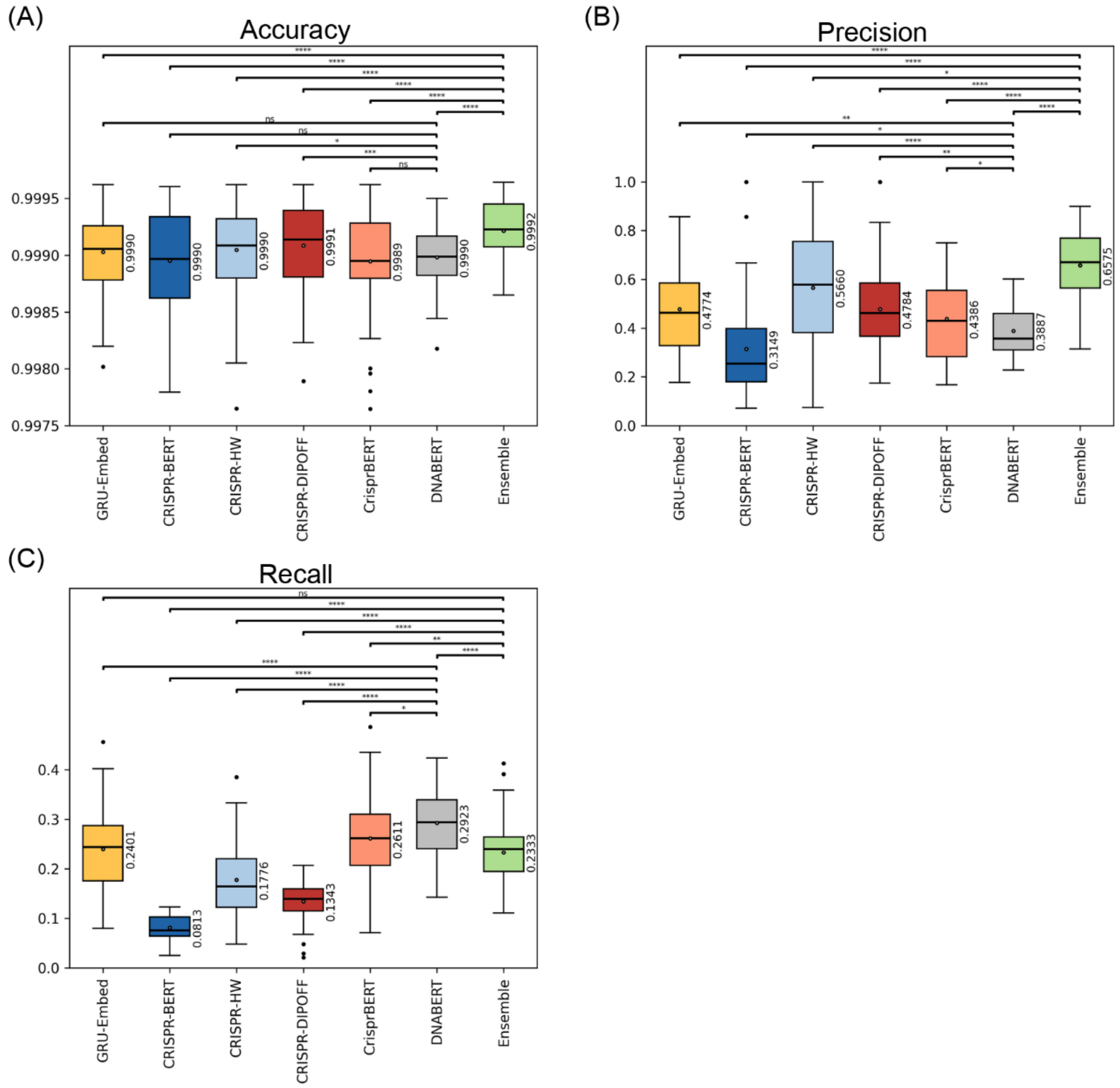

S1 Fig4. Performance comparison of all models on the Schmid-Burgk *et al*. (2020) TTISS dataset (1).

Boxplots show the distribution of (A) Accuracy, (B) Precision, and (C) Recall scores from the cross-validation experiments. The central line in each box indicates the median, the box represents the interquartile range (IQR), and the whiskers extend to 1.5 times the IQR. Dots beyond the whiskers are outliers. Statistical significance between model pairs was determined using the two-sided Wilcoxon signed-rank test with Benjamini-Hochberg correction. Significance levels are denoted as follows: ns: $p > 0.05$, *: $p \leq 0.05$, **: $p \leq 0.01$, ***: $p \leq 0.001$, ****: $p \leq 0.0001$.

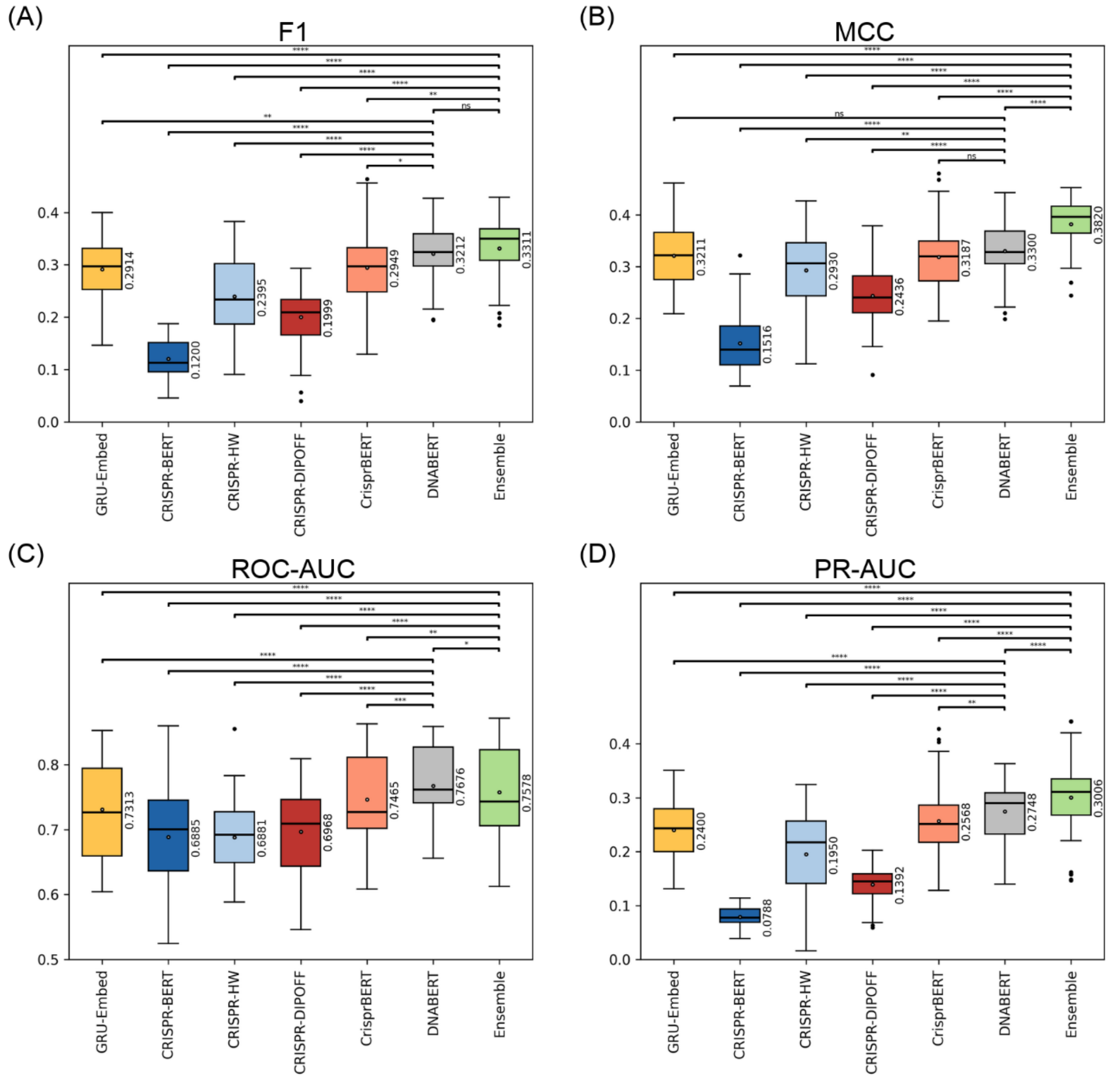

S1 Fig5. Performance comparison of all models on the Schmid-Burgk *et al.* (2020) TTISS dataset (2).

Boxplots show the distribution of (A) F1-score, (B) MCC, (C) ROC-AUC, and (D) PR-AUC scores from the cross-validation experiments. The central line in each box indicates the median, the box represents the interquartile range (IQR), and the whiskers extend to 1.5 times the IQR. Dots beyond the whiskers are outliers. Statistical significance between model pairs was determined using the two-sided Wilcoxon signed-rank test with Benjamini-Hochberg correction. Significance levels are denoted as follows: ns: $p > 0.05$, *: $p \leq 0.05$, **: $p \leq 0.01$, ***: $p \leq 0.001$, ****: $p \leq 0.0001$.

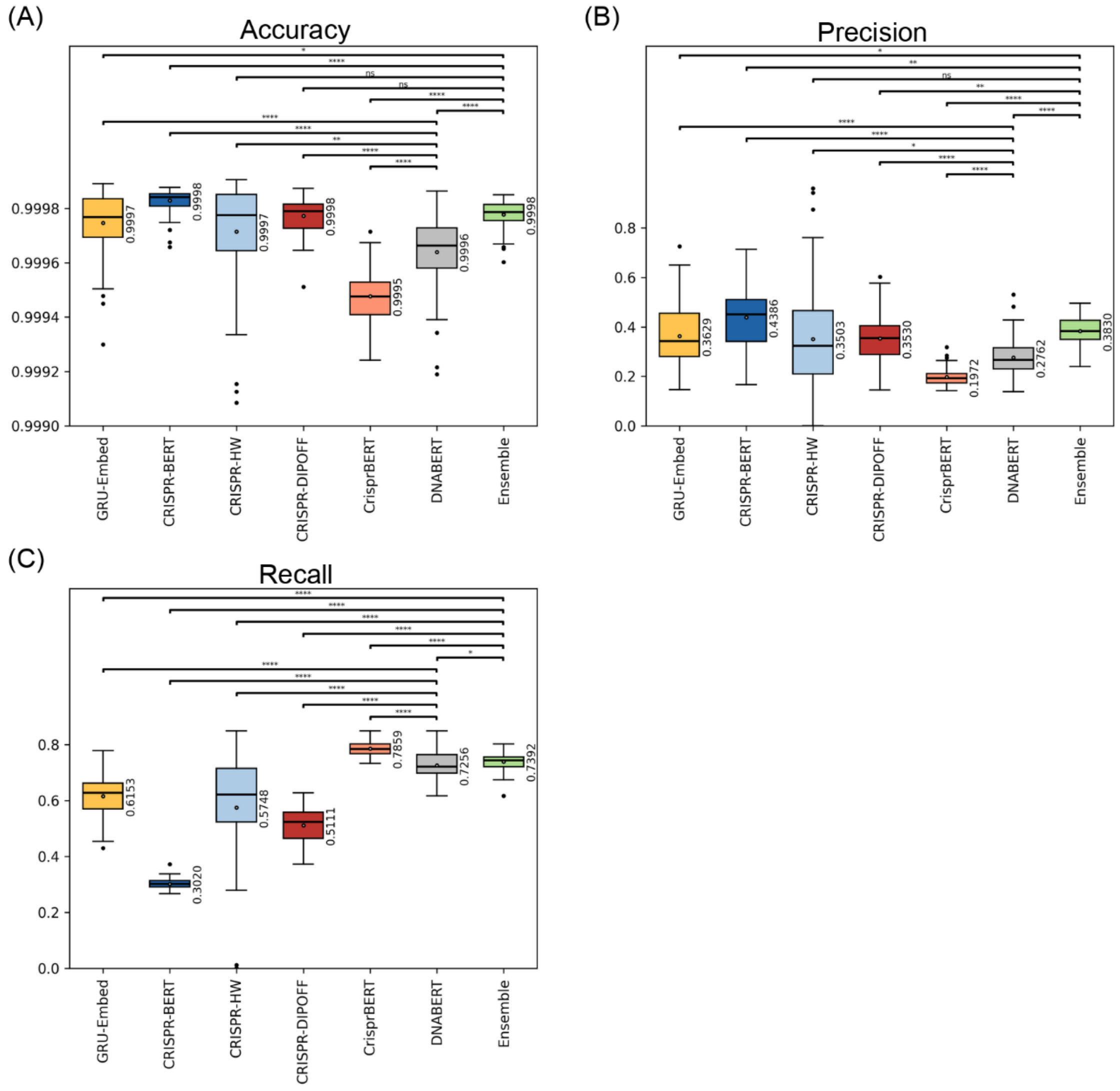

S1 Fig6. Performance comparison of all models on the Listgarten *et al*. (2018) GUIDE-seq dataset (1).

Boxplots show the distribution of (A) Accuracy, (B) Precision, and (C) Recall scores from the cross-validation experiments. The central line in each box indicates the median, the box represents the interquartile range (IQR), and the whiskers extend to 1.5 times the IQR. Dots beyond the whiskers are outliers. Statistical significance between model pairs was determined using the two-sided Wilcoxon signed-rank test with Benjamini-Hochberg correction. Significance levels are denoted as follows: ns: $p > 0.05$, *: $p \leq 0.05$, **: $p \leq 0.01$, ***: $p \leq 0.001$, ****: $p \leq 0.0001$.

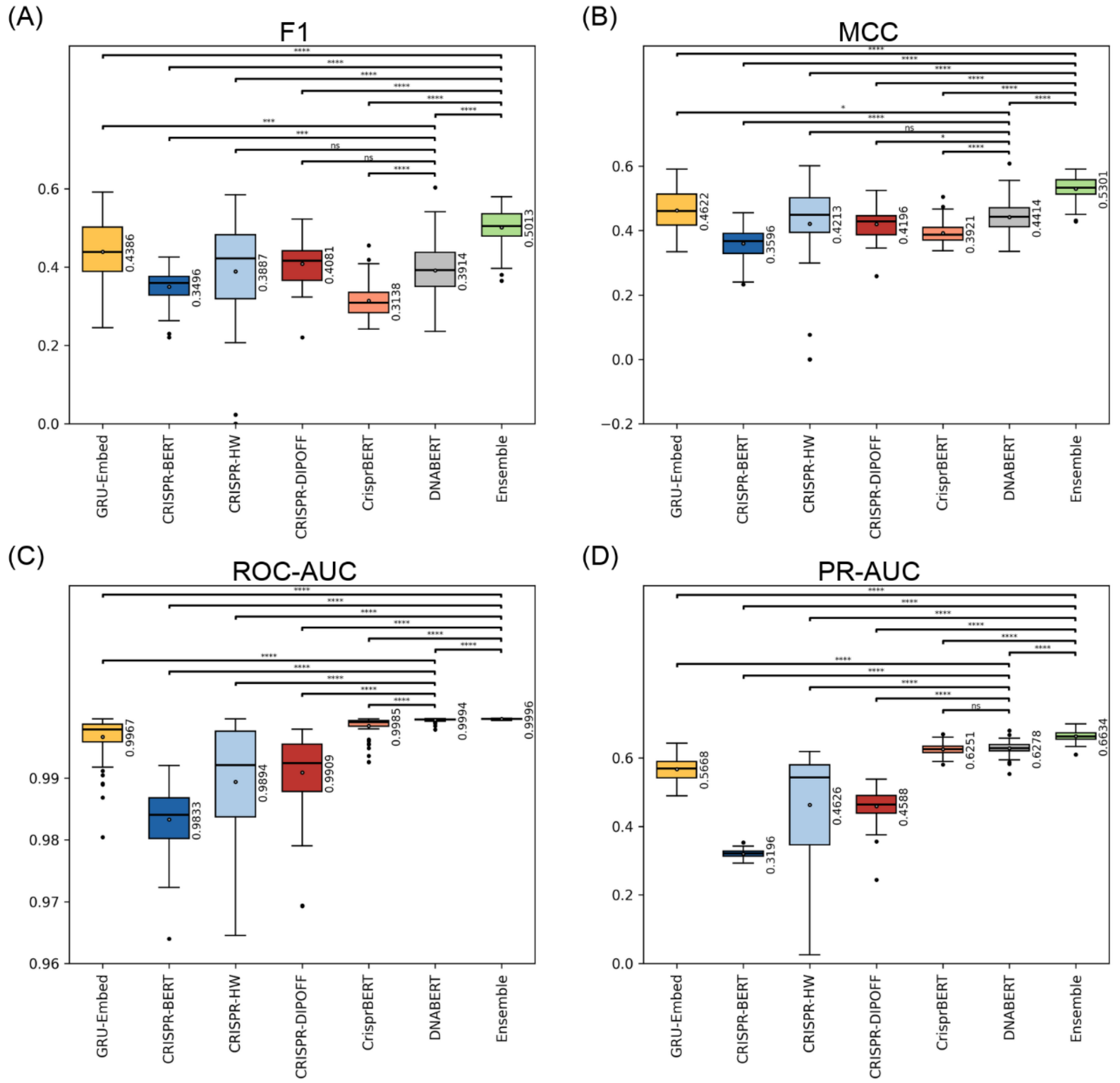

S1 Fig7. Performance comparison of all models on the Listgarten *et al*. (2018) GUIDE-seq dataset (2).

Boxplots show the distribution of (A) F1-score, (B) MCC, (C) ROC-AUC, and (D) PR-AUC scores from the cross-validation experiments. The central line in each box indicates the median, the box represents the interquartile range (IQR), and the whiskers extend to 1.5 times the IQR. Dots beyond the whiskers are outliers. Statistical significance between model pairs was determined using the two-sided Wilcoxon signed-rank test with Benjamini-Hochberg correction. Significance levels are denoted as follows: ns: $p > 0.05$, *: $p \leq 0.05$, **: $p \leq 0.01$, ***: $p \leq 0.001$, ****: $p \leq 0.0001$.

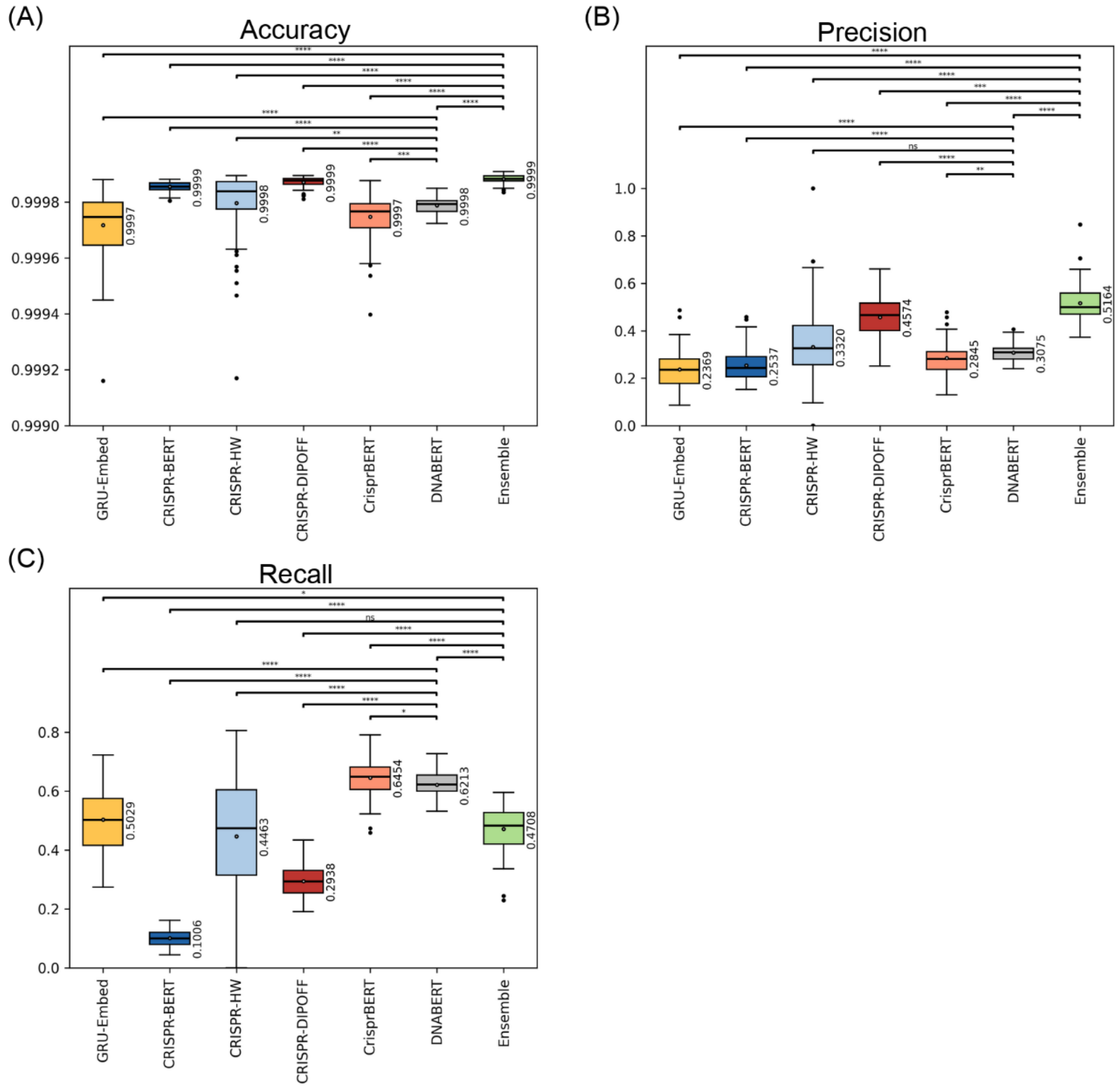

S1 Fig8. Performance comparison of all models on the Chen *et al*. (2017) GUIDE-seq dataset (1).

Boxplots show the distribution of (A) Accuracy, (B) Precision, and (C) Recall scores from the cross-validation experiments. The central line in each box indicates the median, the box represents the interquartile range (IQR), and the whiskers extend to 1.5 times the IQR. Dots beyond the whiskers are outliers. Statistical significance between model pairs was determined using the two-sided Wilcoxon signed-rank test with Benjamini-Hochberg correction. Significance levels are denoted as follows: ns: $p > 0.05$, *: $p \leq 0.05$, **: $p \leq 0.01$, ***: $p \leq 0.001$, ****: $p \leq 0.0001$.

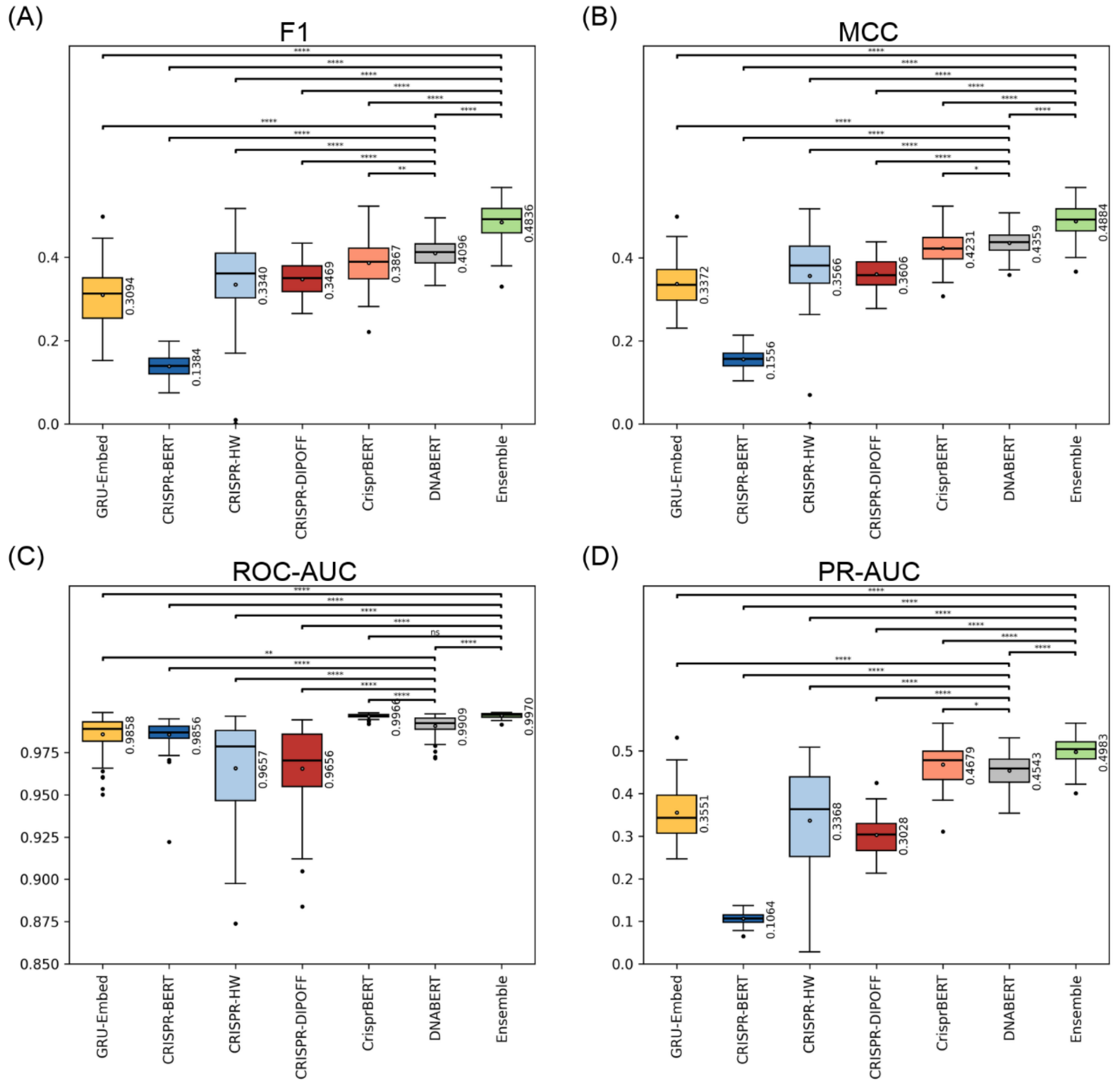

S1 Fig9. Performance comparison of all models on the Chen *et al*. (2017) GUIDE-seq dataset (2).

Boxplots show the distribution of (A) F1-score, (B) MCC, (C) ROC-AUC, and (D) PR-AUC scores from the cross-validation experiments. The central line in each box indicates the median, the box represents the interquartile range (IQR), and the whiskers extend to 1.5 times the IQR. Dots beyond the whiskers are outliers. Statistical significance between model pairs was determined using the two-sided Wilcoxon signed-rank test with Benjamini-Hochberg correction. Significance levels are denoted as follows: ns: $p > 0.05$, *: $p \le 0.05$, **: $p \le 0.01$, ***: $p \le 0.001$, ****: $p \le 0.0001$.

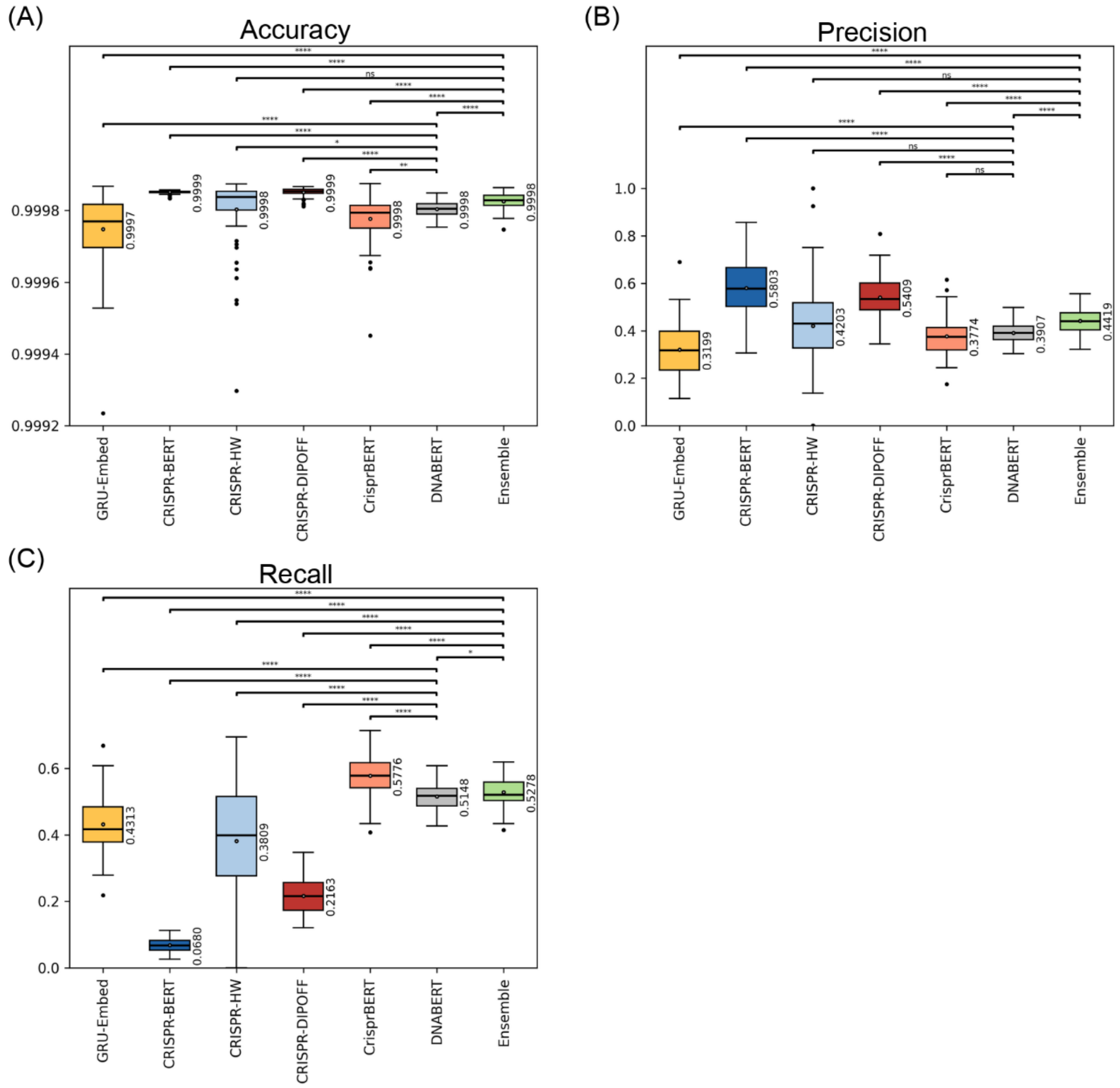

S1 Fig10. Performance comparison of all models on the Tsai *et al.* (2015) GUIDE-seq U2OS dataset (1).

Boxplots show the distribution of (A) Accuracy, (B) Precision, and (C) Recall scores from the cross-validation experiments. The central line in each box indicates the median, the box represents the interquartile range (IQR), and the whiskers extend to 1.5 times the IQR. Dots beyond the whiskers are outliers. Statistical significance between model pairs was determined using the two-sided Wilcoxon signed-rank test with Benjamini-Hochberg correction. Significance levels are denoted as follows: ns: $p > 0.05$, *: $p \le 0.05$, **: $p \le 0.01$, ***: $p \le 0.001$, ****: $p \le 0.0001$.

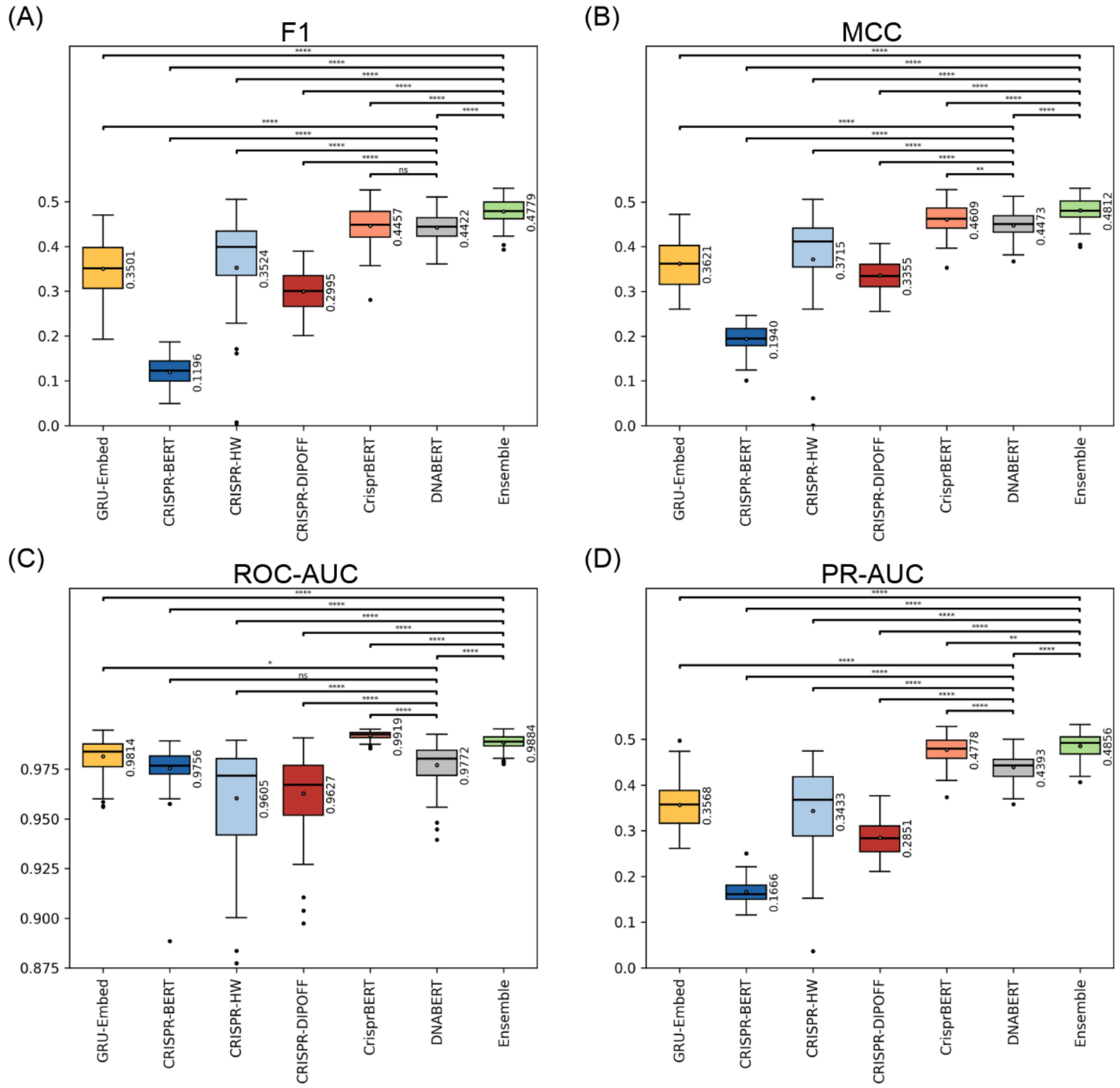

S1 Fig11. Performance comparison of all models on the Tsai *et al.* (2015) GUIDE-seq U2OS dataset (2).

Boxplots show the distribution of (A) F1-score, (B) MCC, (C) ROC-AUC, and (D) PR-AUC scores from the cross-validation experiments. The central line in each box indicates the median, the box represents the interquartile range (IQR), and the whiskers extend to 1.5 times the IQR. Dots beyond the whiskers are outliers. Statistical significance between model pairs was determined using the two-sided Wilcoxon signed-rank test with Benjamini-Hochberg correction. Significance levels are denoted as follows: ns: $p > 0.05$, *: $p \leq 0.05$, **: $p \leq 0.01$, ***: $p \leq 0.001$, ****: $p \leq 0.0001$.

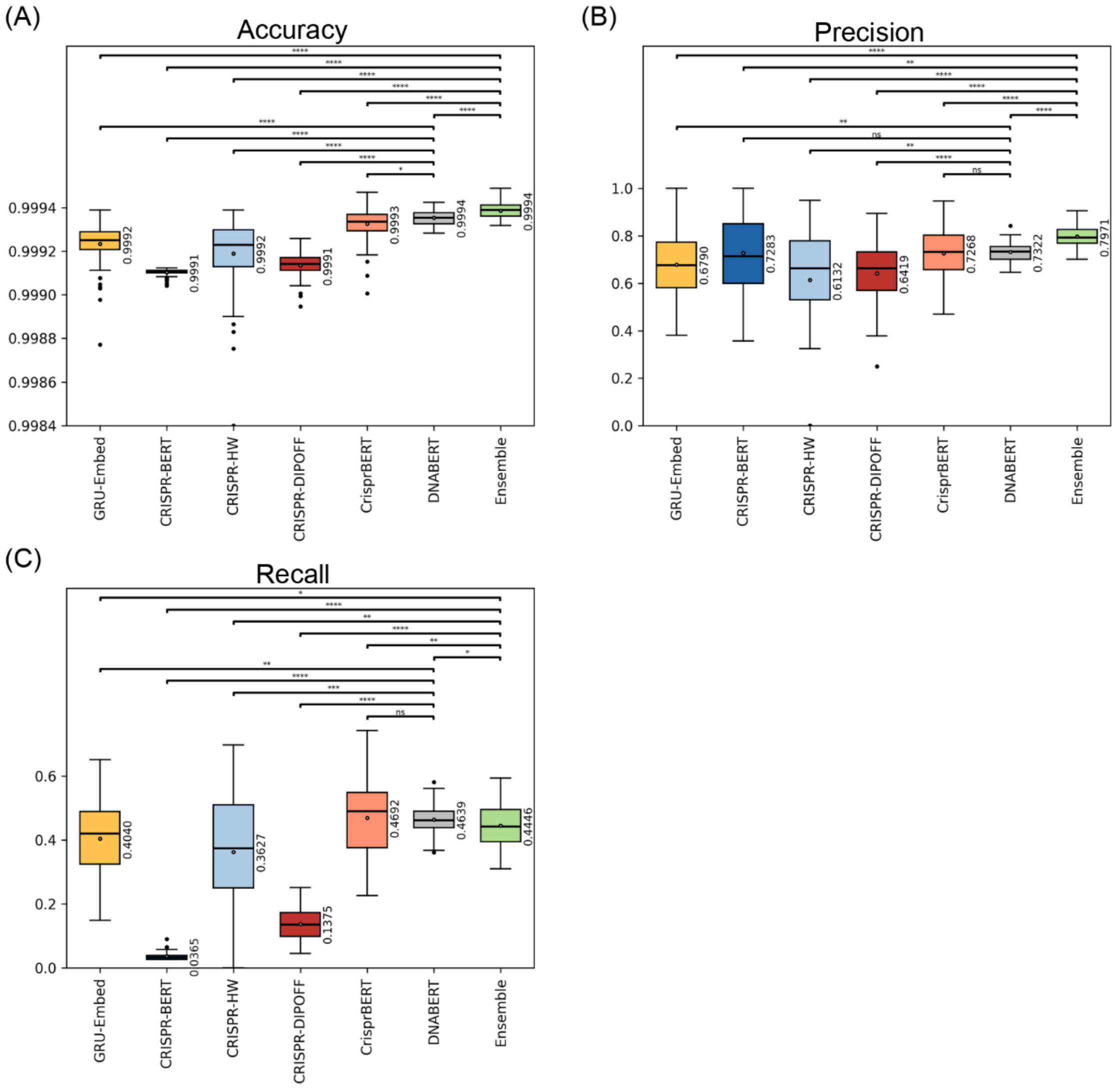

S1 Fig12. Performance comparison of all models on the Tsai *et al*. (2015) GUIDE-seq HEK293 dataset (1).

Boxplots show the distribution of (A) Accuracy, (B) Precision, and (C) Recall scores from the cross-validation experiments. The central line in each box indicates the median, the box represents the interquartile range (IQR), and the whiskers extend to 1.5 times the IQR. Dots beyond the whiskers are outliers. Statistical significance between model pairs was determined using the two-sided Wilcoxon signed-rank test with Benjamini-Hochberg correction. Significance levels are denoted as follows: ns: $p > 0.05$, *: $p \leq 0.05$, **: $p \leq 0.01$, ***: $p \leq 0.001$, ****: $p \leq 0.0001$.

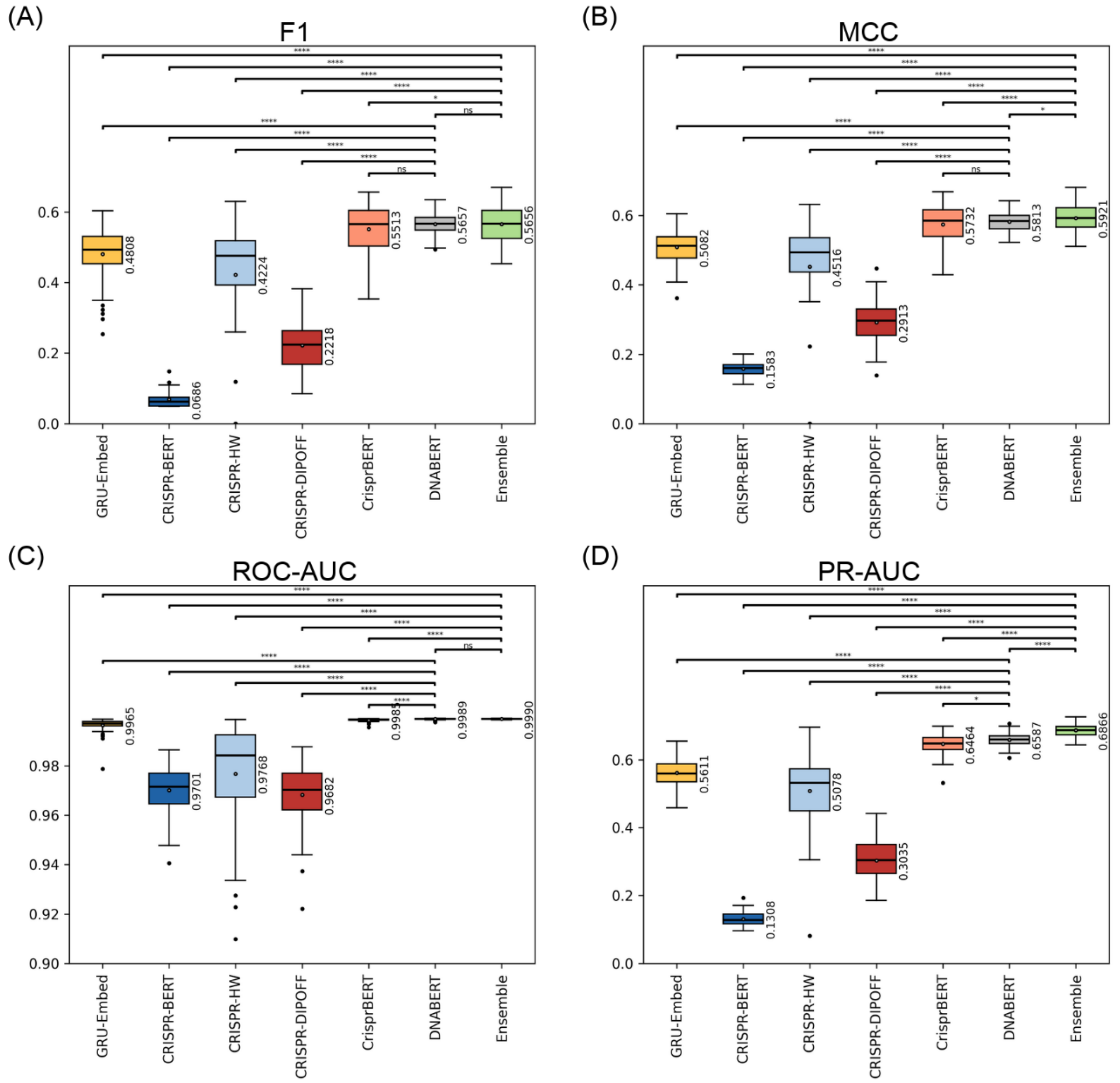

S1 Fig13. Performance comparison of all models on the Tsai *et al.* (2015) GUIDE-seq HEK293 dataset (2).

Boxplots show the distribution of (A) F1-score, (B) MCC, (C) ROC-AUC, and (D) PR-AUC scores from the cross-validation experiments. The central line in each box indicates the median, the box represents the interquartile range (IQR), and the whiskers extend to 1.5 times the IQR. Dots beyond the whiskers are outliers. Statistical significance between model pairs was determined using the two-sided Wilcoxon signed-rank test with Benjamini-Hochberg correction. Significance levels are denoted as follows: ns: $p > 0.05$, *: $p \leq 0.05$, **: $p \leq 0.01$, ***: $p \leq 0.001$, ****: $p \leq 0.0001$.
