# Supplementary material for: Improved CRISPR/Cas9 off-target prediction with DNABERT and epigenetic features
Source: PLoS One. 2025 Nov 12;20(11):e0335863. doi: 10.1371/journal.pone.0335863 (PMC12611124; doi:10.1371/journal.pone.0335863)
Supplement: S2 File — (PDF) [file pone.0335863.s002.pdf]

Supplementary Figures 2: Detailed Performance Comparison of All Models Across All Datasets

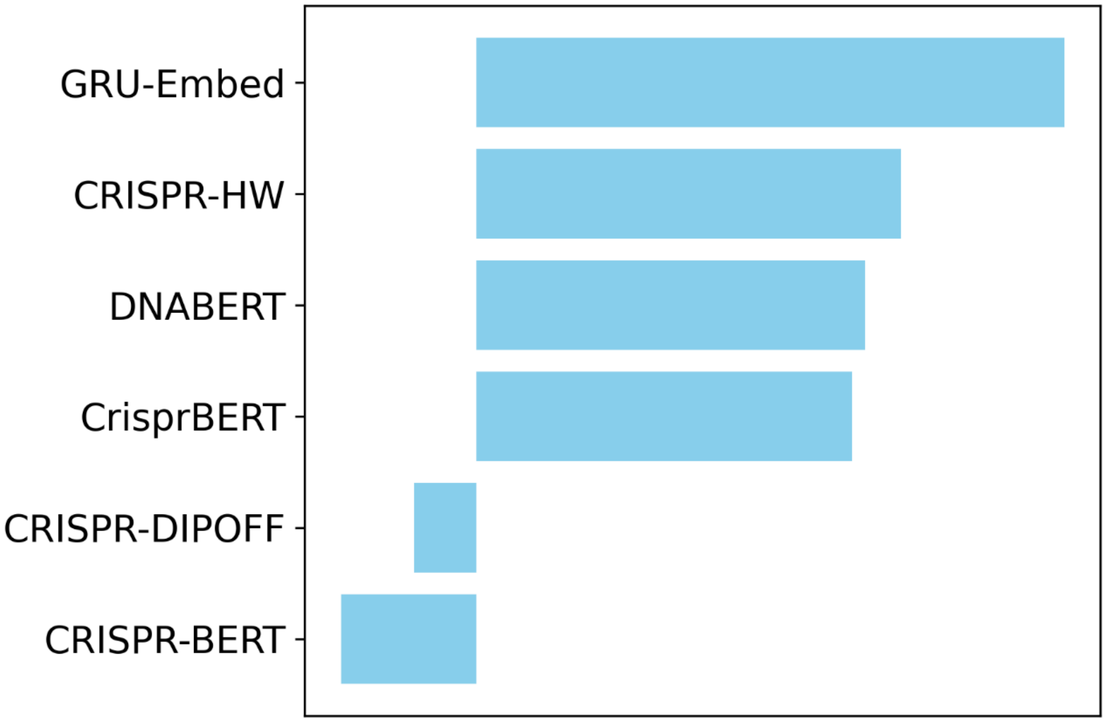

S2 Fig1. Influence of constituent models on ensemble performance for the Lazzarotto *et al*. (2020) CHANGE-Seq dataset.

The contribution of each individual model to the final ensemble was assessed using a leave-one-out approach. Each bar represents the decrease in the ensemble's PR-AUC score when that specific model is excluded from the soft-voting process. A larger bar indicates a greater positive contribution to the ensemble's predictive power.

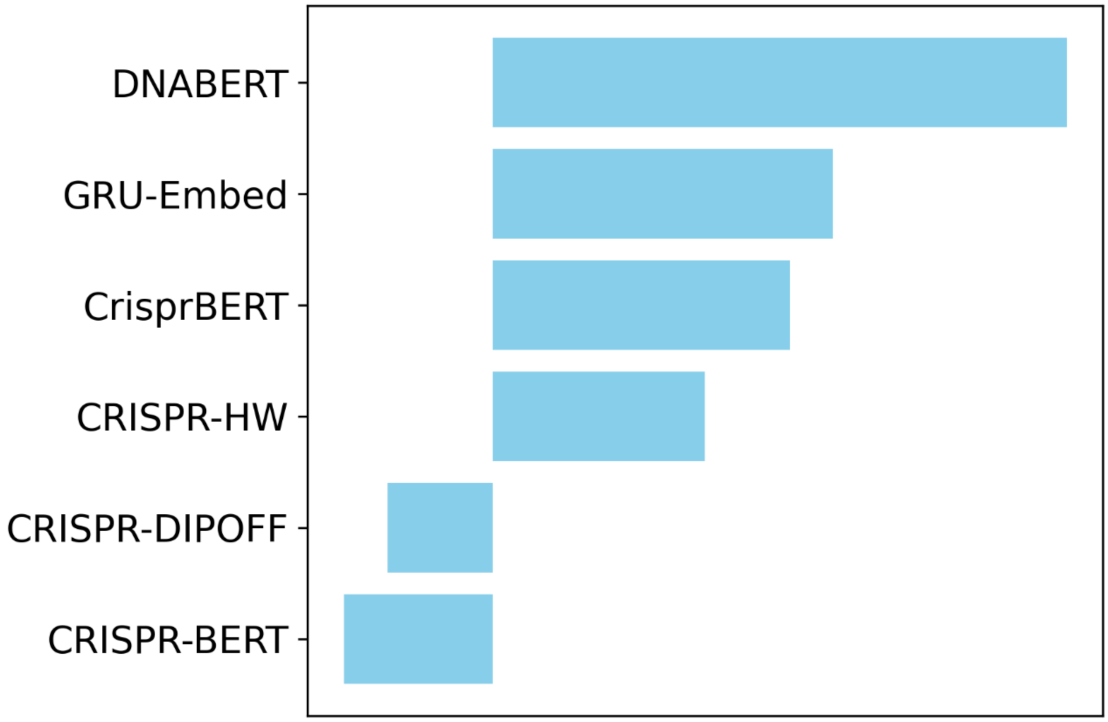

S2 Fig2. Influence of constituent models on ensemble performance for the Schmid-Burgk *et al*. (2020) TTISS dataset.

The contribution of each individual model to the final ensemble was assessed using a leave-one-out approach. Each bar represents the decrease in the ensemble's PR-AUC score when that specific model is excluded from the soft-voting process. A larger bar indicates a greater positive contribution to the ensemble's predictive power.

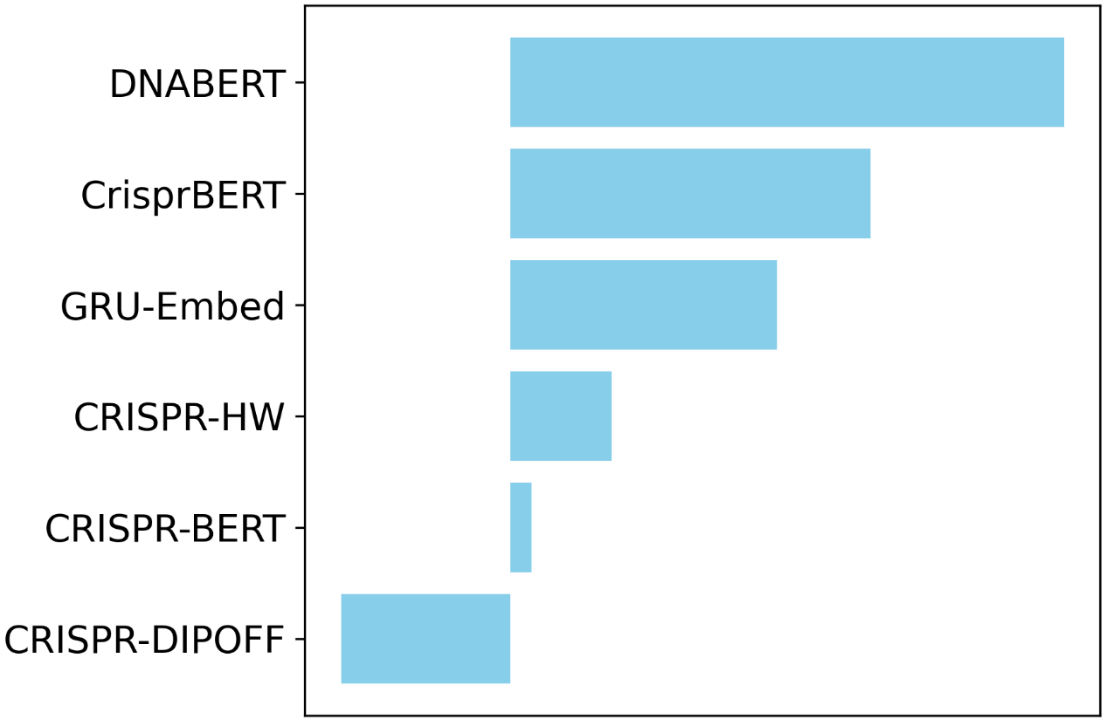

S2 Fig3. Influence of constituent models on ensemble performance for the Listgarten *et al*. (2018) GUIDE-seq dataset.

The contribution of each individual model to the final ensemble was assessed using a leave-one-out approach. Each bar represents the decrease in the ensemble's PR-AUC score when that specific model is excluded from the soft-voting process. A larger bar indicates a greater positive contribution to the ensemble's predictive power.

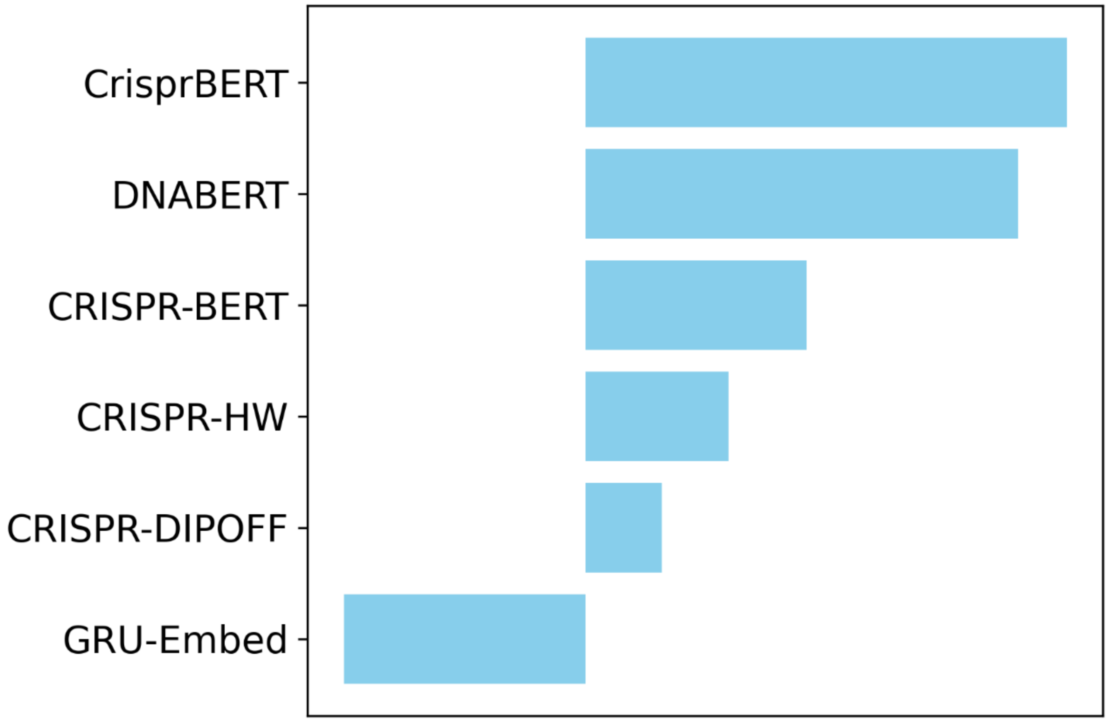

S2 Fig4. Influence of constituent models on ensemble performance for the Chen *et al*. (2017) GUIDE-seq dataset.

The contribution of each individual model to the final ensemble was assessed using a leave-one-out approach. Each bar represents the decrease in the ensemble's PR-AUC score when that specific model is excluded from the soft-voting process. A larger bar indicates a greater positive contribution to the ensemble's predictive power.

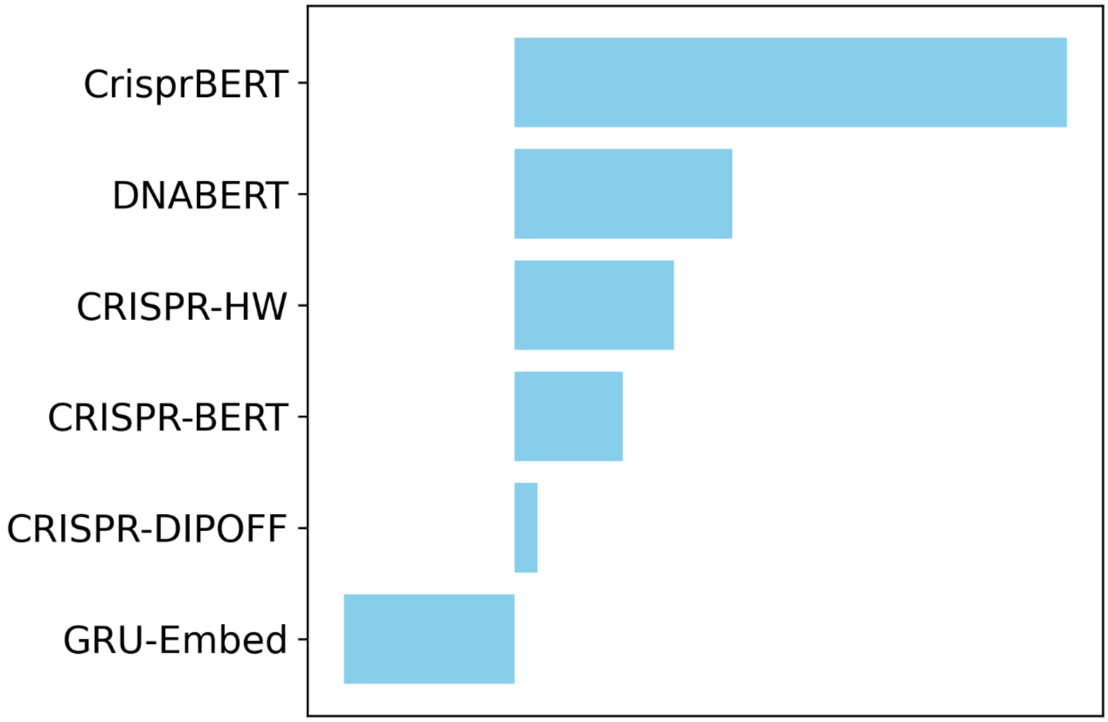

S2 Fig5. Influence of constituent models on ensemble performance for the Tsai *et al*. (2015) GUIDE-seq U2OS dataset.

The contribution of each individual model to the final ensemble was assessed using a leave-one-out approach. Each bar represents the decrease in the ensemble's PR-AUC score when that specific model is excluded from the soft-voting process. A larger bar indicates a greater positive contribution to the ensemble's predictive power.

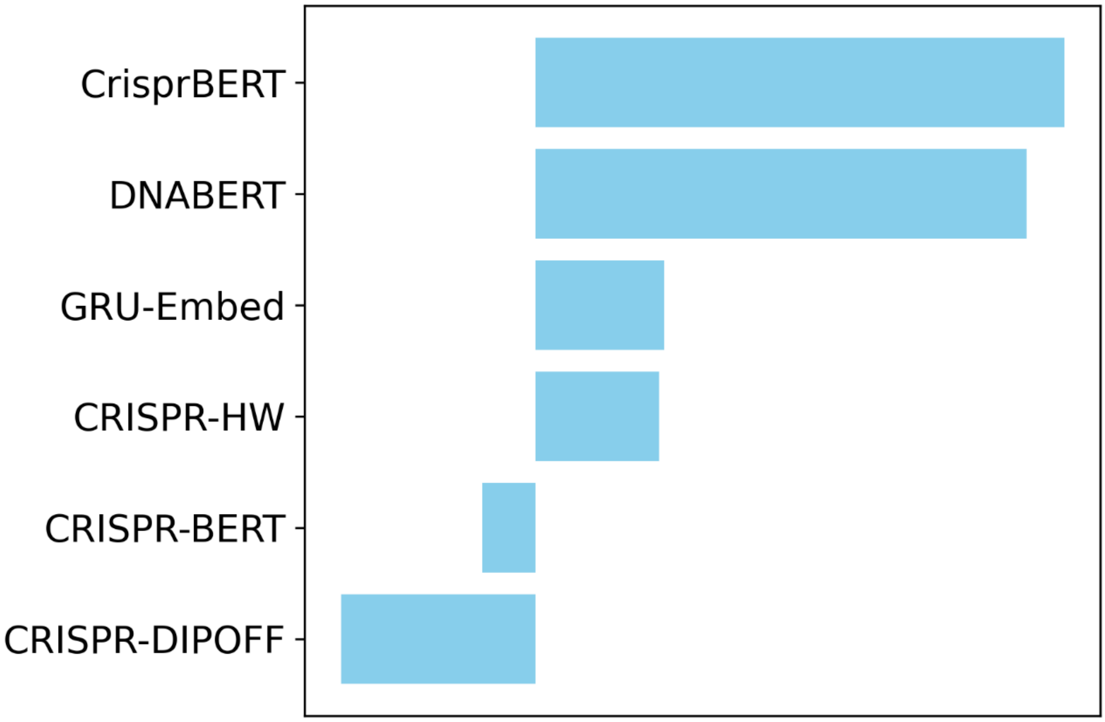

S2 Fig6. Influence of constituent models on ensemble performance for the Tsai *et al*. (2015) GUIDE-seq HEK293 dataset.

The contribution of each individual model to the final ensemble was assessed using a leave-one-out approach. Each bar represents the decrease in the ensemble's PR-AUC score when that specific model is excluded from the soft-voting process. A larger bar indicates a greater positive contribution to the ensemble's predictive power.
