# Supplementary material for: Improved CRISPR/Cas9 off-target prediction with DNABERT and epigenetic features
Source: PLoS One. 2025 Nov 12;20(11):e0335863. doi: 10.1371/journal.pone.0335863 (PMC12611124; doi:10.1371/journal.pone.0335863)
Supplement: S3 File — (PDF) [file pone.0335863.s003.pdf]

Supplementary Figures 3: SHAP Summary Plots

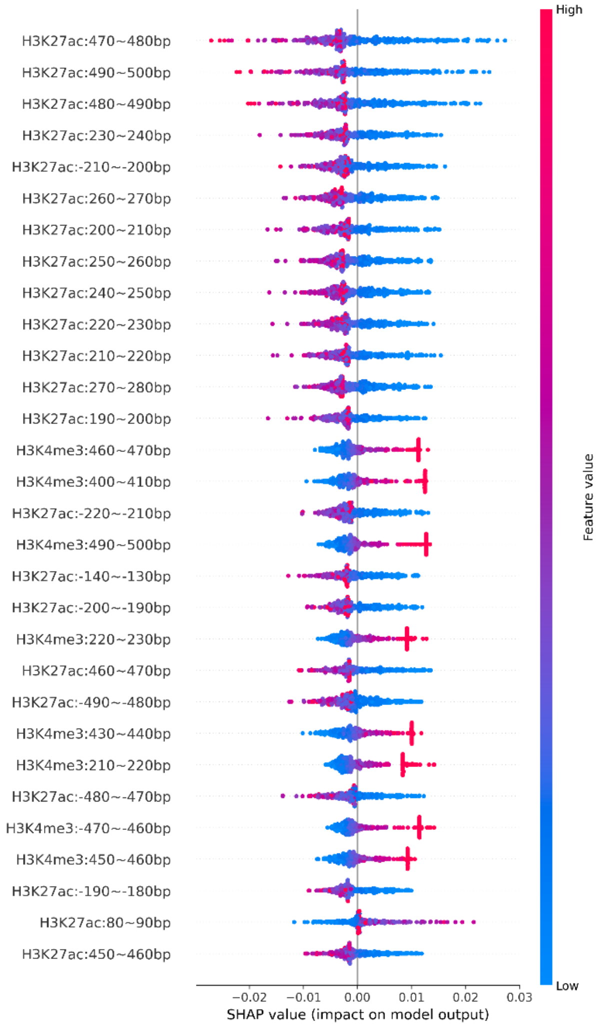

S3 Fig1. SHAP summary plot for the DNABERT-Epi model from cross-validation fold 1st of 14.

This plot illustrates the contribution of the top epigenetic features to the model's prediction for the positive class (active off-target) on the Lazzarotto *et al*. (2020) GUIDE-seq dataset. Features on the y-axis are ranked by their global importance (mean absolute SHAP value). Each point represents the SHAP value for a feature in a single sample; its position on the x-axis indicates the impact on the model's log-odds output. The color corresponds to the feature value, with red representing high values and blue representing low values. This plot was generated from the model trained on the 1st fold of the 14-fold cross-validation.

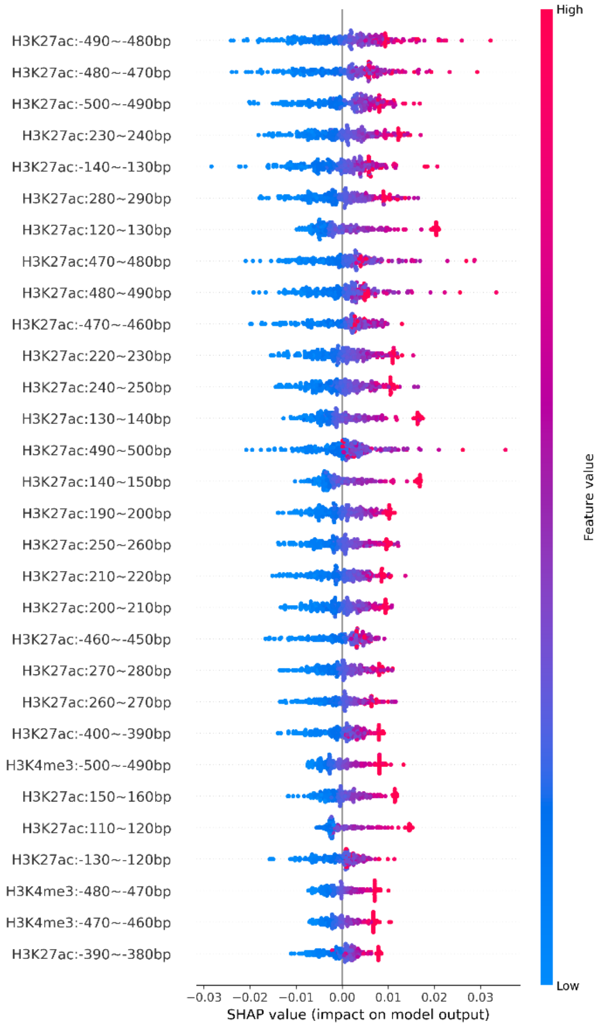

S3 Fig2. SHAP summary plot for the DNABERT-Epi model from cross-validation fold 2nd of 14.

This plot illustrates the contribution of the top epigenetic features to the model's prediction for the positive class (active off-target) on the Lazzarotto *et al*. (2020) GUIDE-seq dataset. Features on the y-axis are ranked by their global importance (mean absolute SHAP value). Each point represents the SHAP value for a feature in a single sample; its position on the x-axis indicates the impact on the model's log-odds output. The color corresponds to the feature value, with red representing high values and blue representing low values. This plot was generated from the model trained on the 2nd fold of the 14-fold cross-validation.

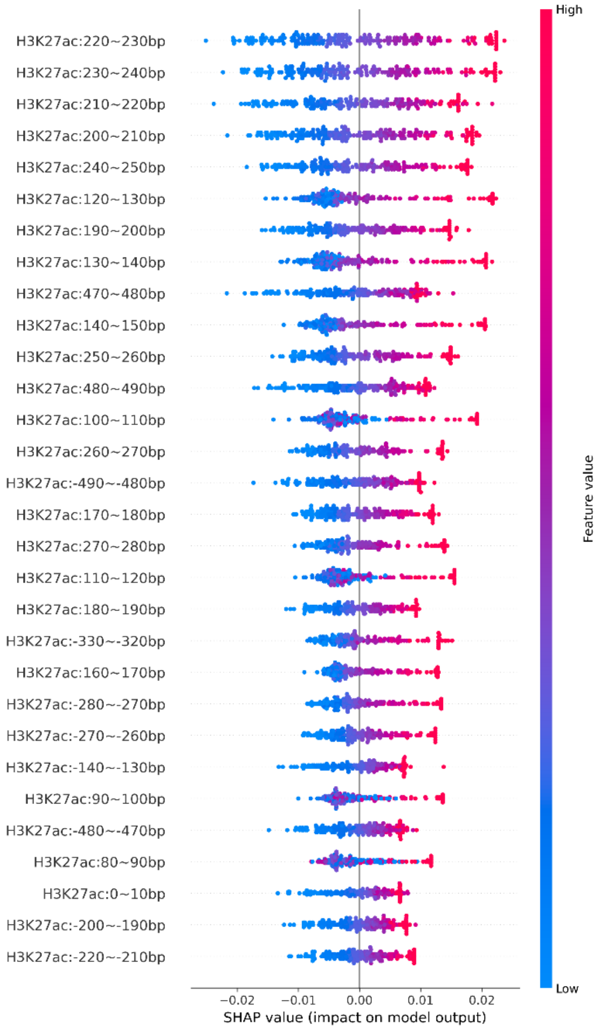

S3 Fig3. SHAP summary plot for the DNABERT-Epi model from cross-validation fold 3rd of 14.

This plot illustrates the contribution of the top epigenetic features to the model's prediction for the positive class (active off-target) on the Lazzarotto *et al*. (2020) GUIDE-seq dataset. Features on the y-axis are ranked by their global importance (mean absolute SHAP value). Each point represents the SHAP value for a feature in a single sample; its position on the x-axis indicates the impact on the model's log-odds output. The color corresponds to the feature value, with red representing high values and blue representing low values. This plot was generated from the model trained on the 3rd fold of the 14-fold cross-validation.

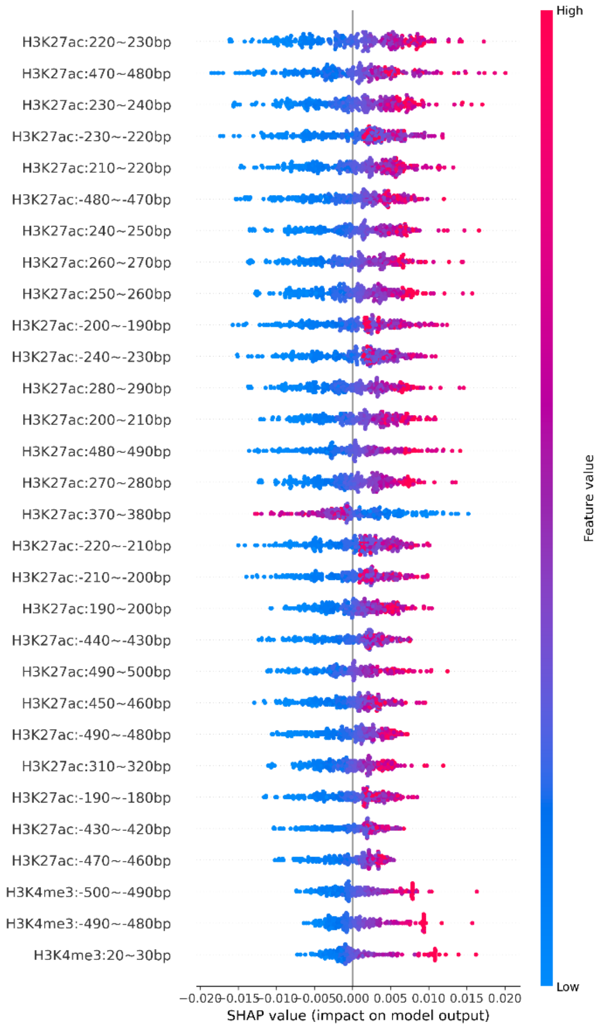

S3 Fig4. SHAP summary plot for the DNABERT-Epi model from cross-validation fold 4th of 14.

This plot illustrates the contribution of the top epigenetic features to the model's prediction for the positive class (active off-target) on the Lazzarotto *et al*. (2020) GUIDE-seq dataset. Features on the y-axis are ranked by their global importance (mean absolute SHAP value). Each point represents the SHAP value for a feature in a single sample; its position on the x-axis indicates the impact on the model's log-odds output. The color corresponds to the feature value, with red representing high values and blue representing low values. This plot was generated from the model trained on the 4th fold of the 14-fold cross-validation.

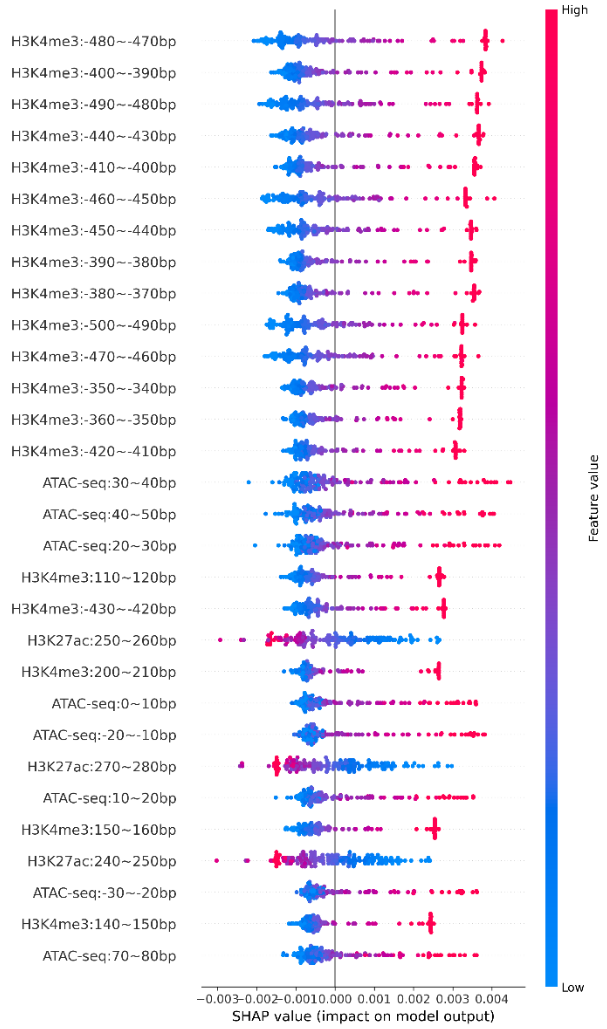

S3 Fig5. SHAP summary plot for the DNABERT-Epi model from cross-validation fold 5th of 14.

This plot illustrates the contribution of the top epigenetic features to the model's prediction for the positive class (active off-target) on the Lazzarotto *et al*. (2020) GUIDE-seq dataset. Features on the y-axis are ranked by their global importance (mean absolute SHAP value). Each point represents the SHAP value for a feature in a single sample; its position on the x-axis indicates the impact on the model's log-odds output. The color corresponds to the feature value, with red representing high values and blue representing low values. This plot was generated from the model trained on the 5th fold of the 14-fold cross-validation.

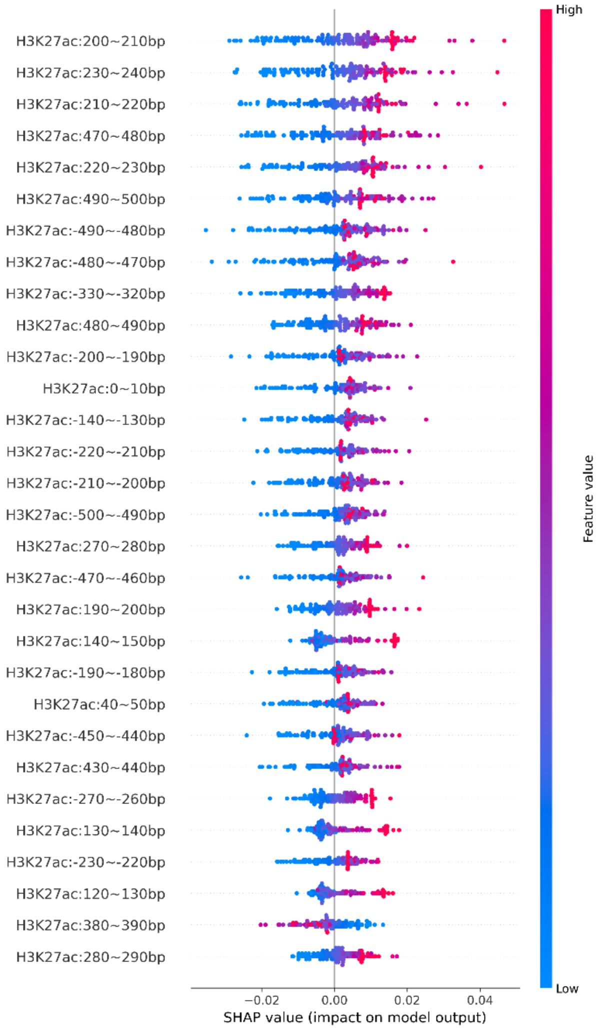

S3 Fig6. SHAP summary plot for the DNABERT-Epi model from cross-validation fold 6th of 14.

This plot illustrates the contribution of the top epigenetic features to the model's prediction for the positive class (active off-target) on the Lazzarotto *et al*. (2020) GUIDE-seq dataset. Features on the y-axis are ranked by their global importance (mean absolute SHAP value). Each point represents the SHAP value for a feature in a single sample; its position on the x-axis indicates the impact on the model's log-odds output. The color corresponds to the feature value, with red representing high values and blue representing low values. This plot was generated from the model trained on the 6th fold of the 14-fold cross-validation.

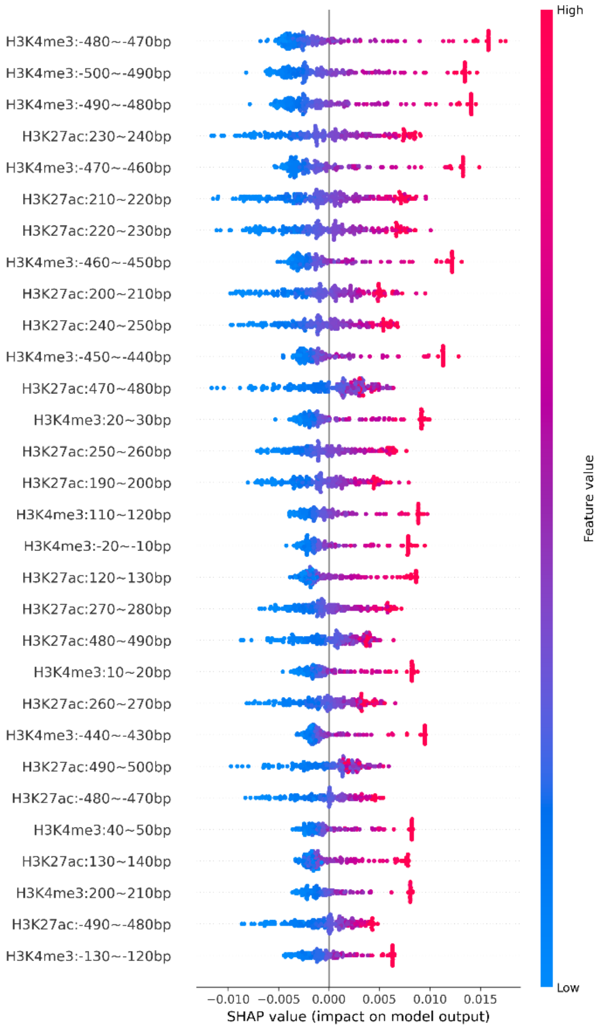

S3 Fig7. SHAP summary plot for the DNABERT-Epi model from cross-validation fold 7th of 14.

This plot illustrates the contribution of the top epigenetic features to the model's prediction for the positive class (active off-target) on the Lazzarotto *et al*. (2020) GUIDE-seq dataset. Features on the y-axis are ranked by their global importance (mean absolute SHAP value). Each point represents the SHAP value for a feature in a single sample; its position on the x-axis indicates the impact on the model's log-odds output. The color corresponds to the feature value, with red representing high values and blue representing low values. This plot was generated from the model trained on the 7th fold of the 14-fold cross-validation.

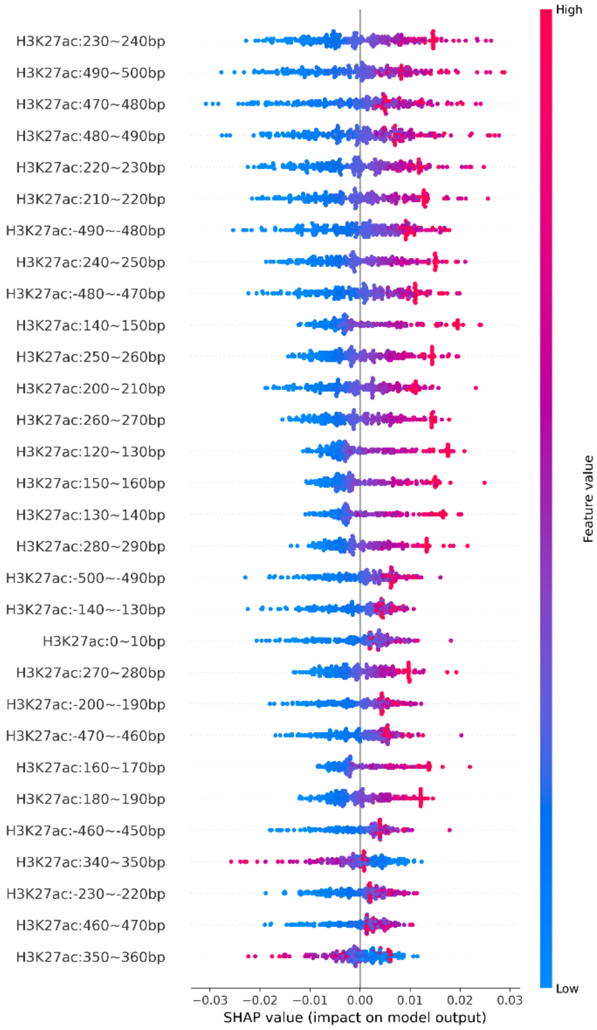

S3 Fig8. SHAP summary plot for the DNABERT-Epi model from cross-validation fold 8th of 14.

This plot illustrates the contribution of the top epigenetic features to the model's prediction for the positive class (active off-target) on the Lazzarotto *et al*. (2020) GUIDE-seq dataset. Features on the y-axis are ranked by their global importance (mean absolute SHAP value). Each point represents the SHAP value for a feature in a single sample; its position on the x-axis indicates the impact on the model's log-odds output. The color corresponds to the feature value, with red representing high values and blue representing low values. This plot was generated from the model trained on the 8th fold of the 14-fold cross-validation.

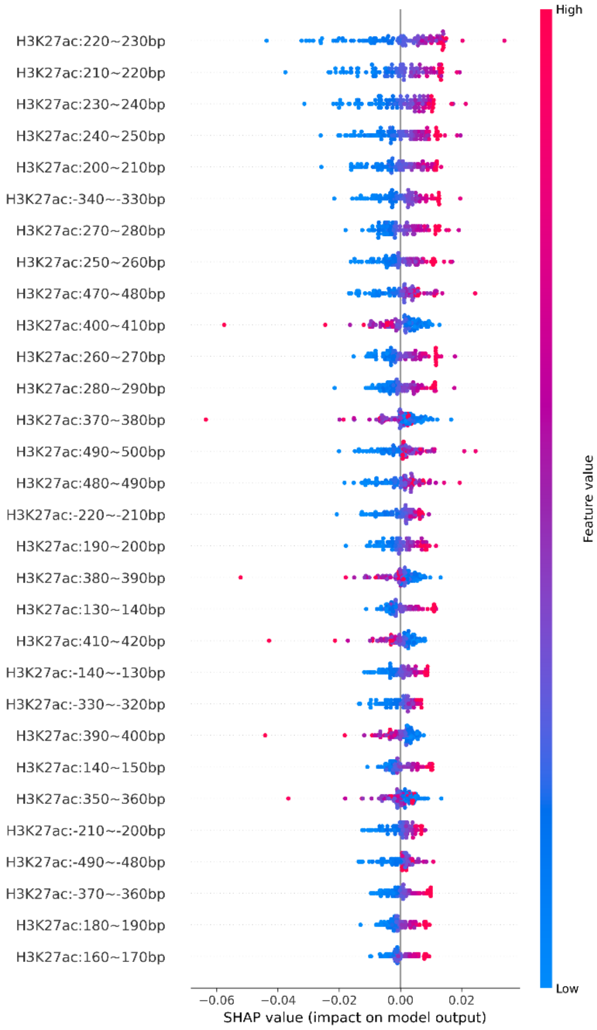

S3 Fig9. SHAP summary plot for the DNABERT-Epi model from cross-validation fold 9th of 14.

This plot illustrates the contribution of the top epigenetic features to the model's prediction for the positive class (active off-target) on the Lazzarotto *et al*. (2020) GUIDE-seq dataset. Features on the y-axis are ranked by their global importance (mean absolute SHAP value). Each point represents the SHAP value for a feature in a single sample; its position on the x-axis indicates the impact on the model's log-odds output. The color corresponds to the feature value, with red representing high values and blue representing low values. This plot was generated from the model trained on the 9th fold of the 14-fold cross-validation.

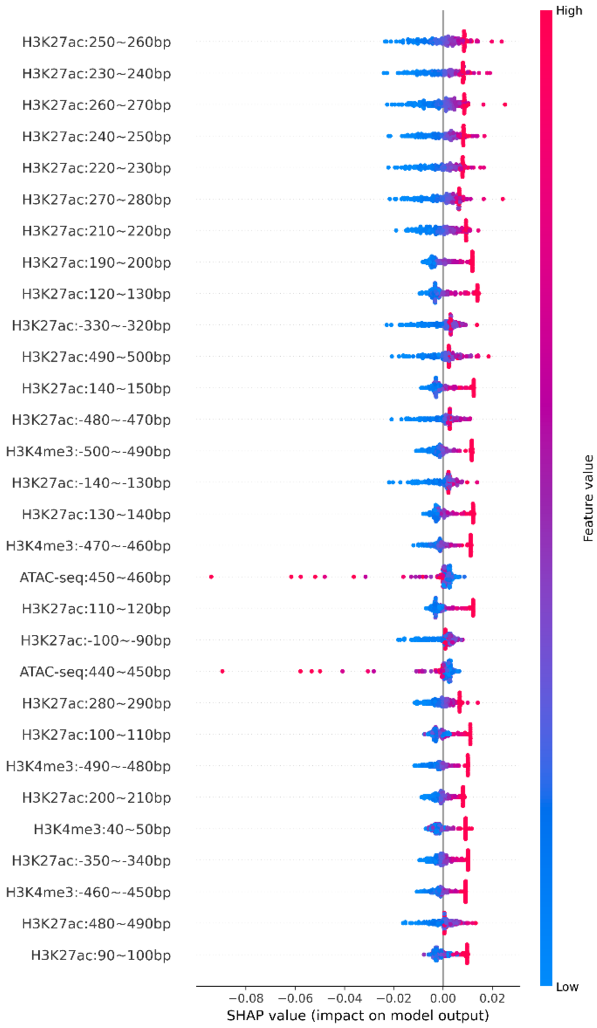

S3 Fig10. SHAP summary plot for the DNABERT-Epi model from cross-validation fold 10th of 14.

This plot illustrates the contribution of the top epigenetic features to the model's prediction for the positive class (active off-target) on the Lazzarotto *et al*. (2020) GUIDE-seq dataset. Features on the y-axis are ranked by their global importance (mean absolute SHAP value). Each point represents the SHAP value for a feature in a single sample; its position on the x-axis indicates the impact on the model's log-odds output. The color corresponds to the feature value, with red representing high values and blue representing low values. This plot was generated from the model trained on the 10th fold of the 14-fold cross-validation.

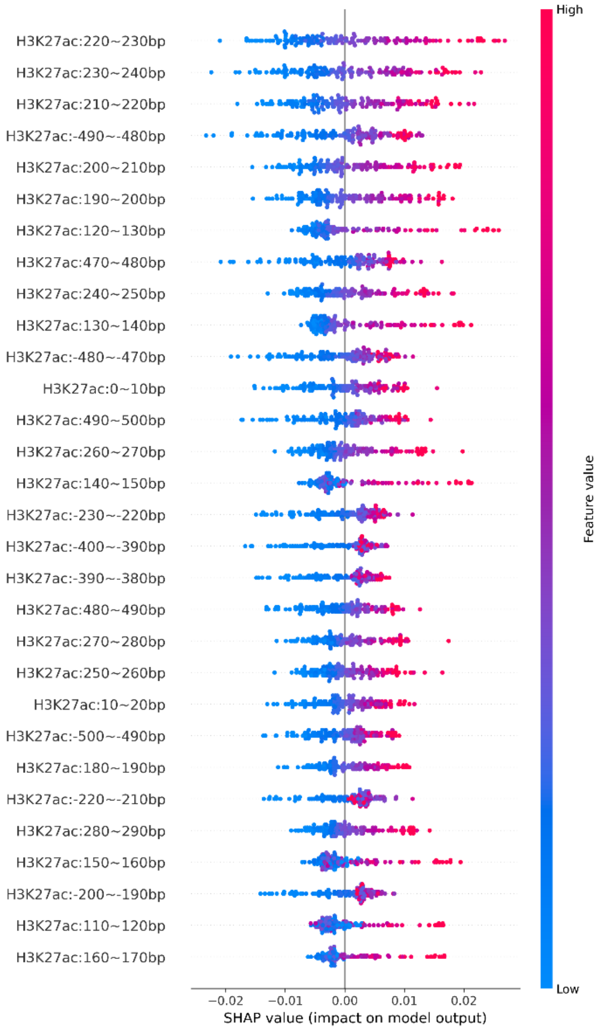

S3 Fig11. SHAP summary plot for the DNABERT-Epi model from cross-validation fold 12th of 14.

This plot illustrates the contribution of the top epigenetic features to the model's prediction for the positive class (active off-target) on the Lazzarotto *et al*. (2020) GUIDE-seq dataset. Features on the y-axis are ranked by their global importance (mean absolute SHAP value). Each point represents the SHAP value for a feature in a single sample; its position on the x-axis indicates the impact on the model's log-odds output. The color corresponds to the feature value, with red representing high values and blue representing low values. This plot was generated from the model trained on the 12th fold of the 14-fold cross-validation.

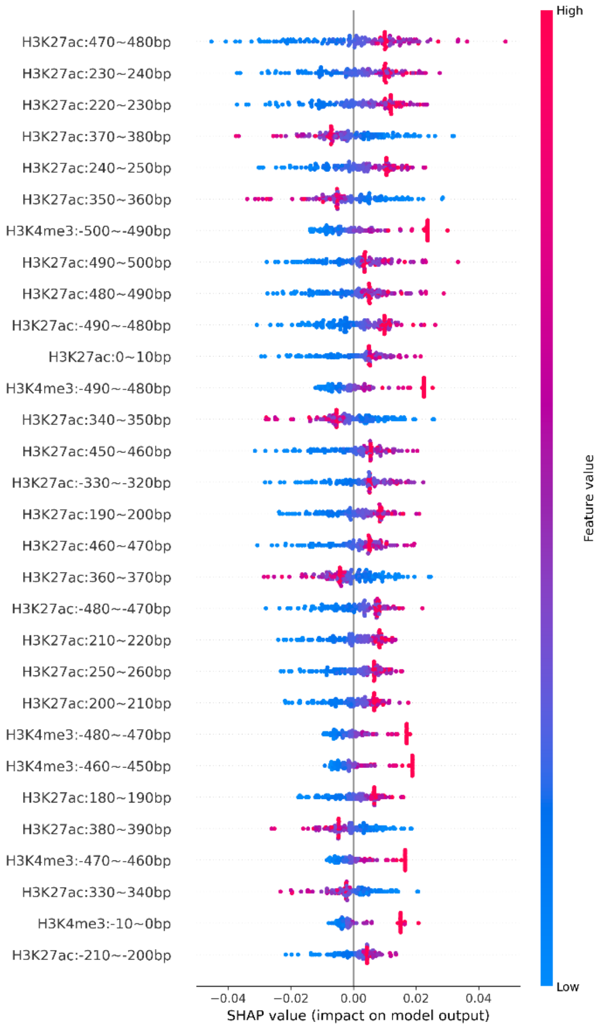

S3 Fig12. SHAP summary plot for the DNABERT-Epi model from cross-validation fold 13th of 14.

This plot illustrates the contribution of the top epigenetic features to the model's prediction for the positive class (active off-target) on the Lazzarotto *et al*. (2020) GUIDE-seq dataset. Features on the y-axis are ranked by their global importance (mean absolute SHAP value). Each point represents the SHAP value for a feature in a single sample; its position on the x-axis indicates the impact on the model's log-odds output. The color corresponds to the feature value, with red representing high values and blue representing low values. This plot was generated from the model trained on the 13th fold of the 14-fold cross-validation.

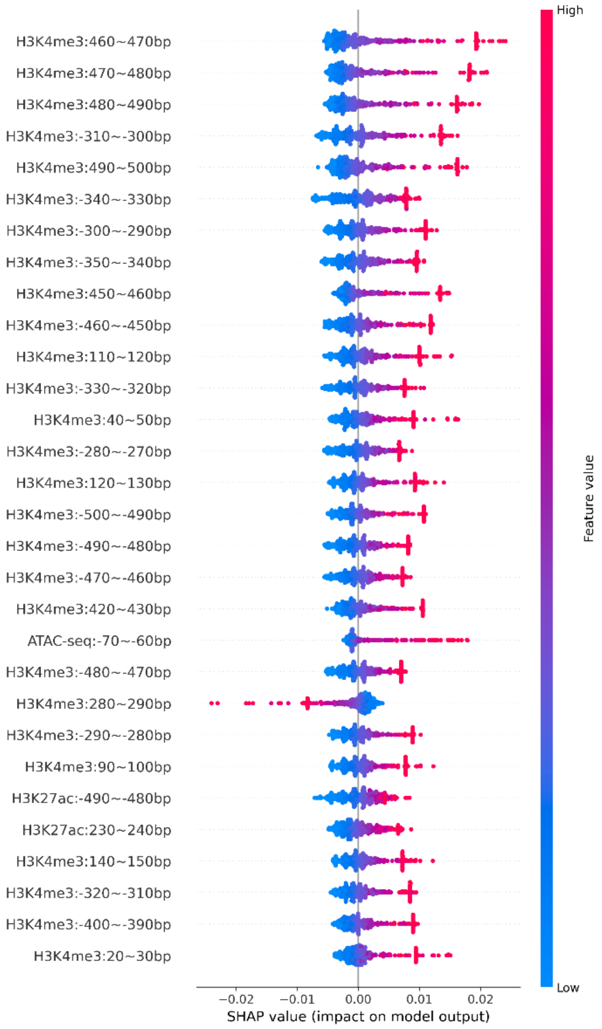

S3 Fig13. SHAP summary plot for the DNABERT-Epi model from cross-validation fold 14th of 14.

This plot illustrates the contribution of the top epigenetic features to the model's prediction for the positive class (active off-target) on the Lazzarotto *et al*. (2020) GUIDE-seq dataset. Features on the y-axis are ranked by their global importance (mean absolute SHAP value). Each point represents the SHAP value for a feature in a single sample; its position on the x-axis indicates the impact on the model's log-odds output. The color corresponds to the feature value, with red representing high values and blue representing low values. This plot was generated from the model trained on the 14th fold of the 14-fold cross-validation.
