# Supplementary material for: Improved CRISPR/Cas9 off-target prediction with DNABERT and epigenetic features
Source: PLoS One. 2025 Nov 12;20(11):e0335863. doi: 10.1371/journal.pone.0335863 (PMC12611124; doi:10.1371/journal.pone.0335863)
Supplement: S4 File — (PDF) [file pone.0335863.s004.pdf]

Supplementary Figures 4: Integrated Gradients Attribution Heatmaps for All Analyzed sgRNAs

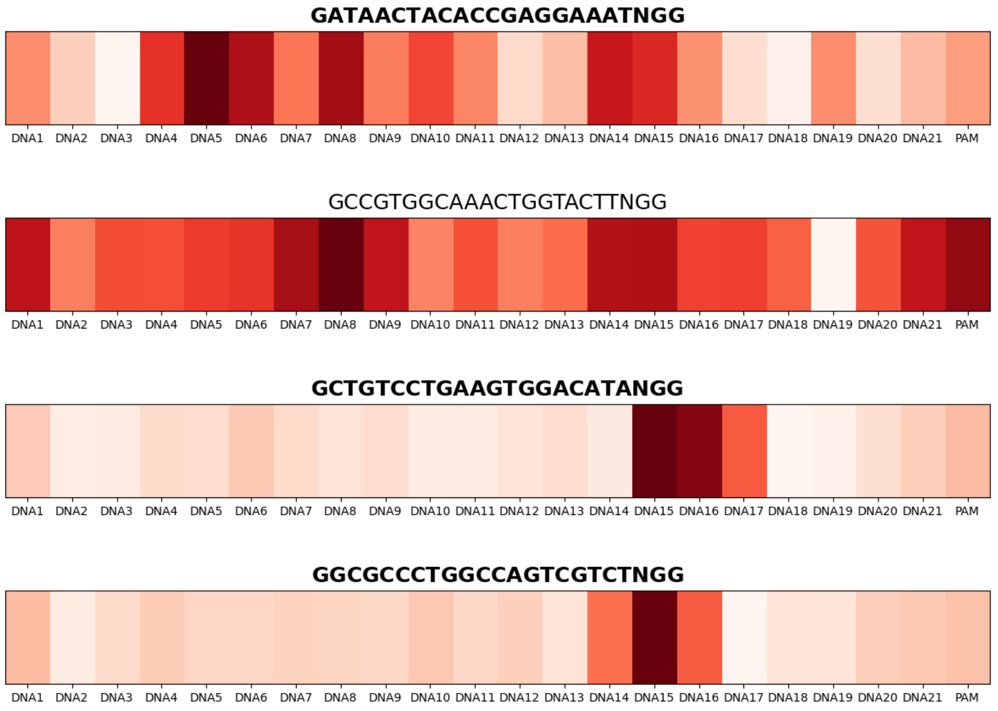

S4 Fig1. Integrated Gradients attribution heatmaps for the Lazzarotto *et al*. (2020) GUIDE-seq dataset from cross-validation fold 1st of 14.

Each row displays a heatmap of attribution scores for a single sgRNA, with its sequence shown above. The columns correspond to the 3-mer token positions in the target DNA sequence, from the first token (DNA1) to the PAM site. Color intensity reflects the attribution score, where darker red indicates a stronger positive contribution to the model's prediction of an off-target event. The sgRNA sequence is displayed in bold if its maximum attribution score is located within the PAM-distal (positions 4–6) or PAM-proximal (positions 13-17) hotspots. This analysis was performed using the DNABERT model trained on the corresponding data from this cross-validation fold.

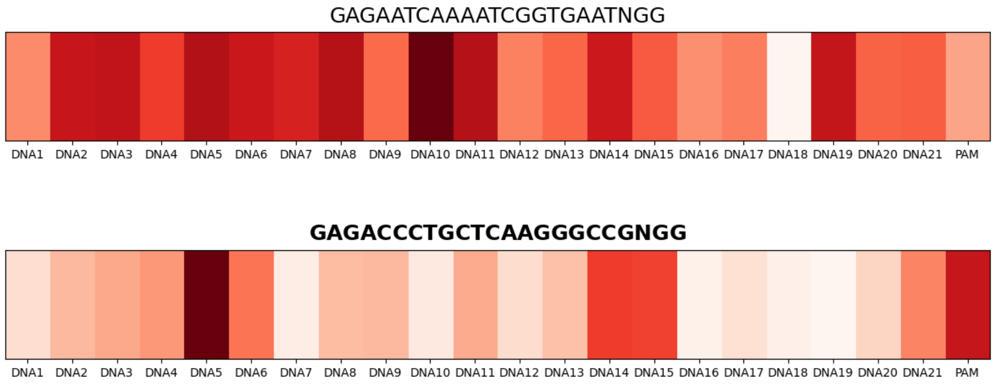

S4 Fig2. Integrated Gradients attribution heatmaps for the Lazzarotto *et al*. (2020) GUIDE-seq dataset from cross-validation fold 2nd of 14.

Each row displays a heatmap of attribution scores for a single sgRNA, with its sequence shown above. The columns correspond to the 3-mer token positions in the target DNA sequence, from the first token (DNA1) to the PAM site. Color intensity reflects the attribution score, where darker red indicates a stronger positive contribution to the model's prediction of an off-target event. The sgRNA sequence is displayed in bold if its maximum attribution score is located within the PAM-distal (positions 4–6) or PAM-proximal (positions 13‐17) hotspots. This analysis was performed using the DNABERT model trained on the corresponding data from this cross-validation fold.

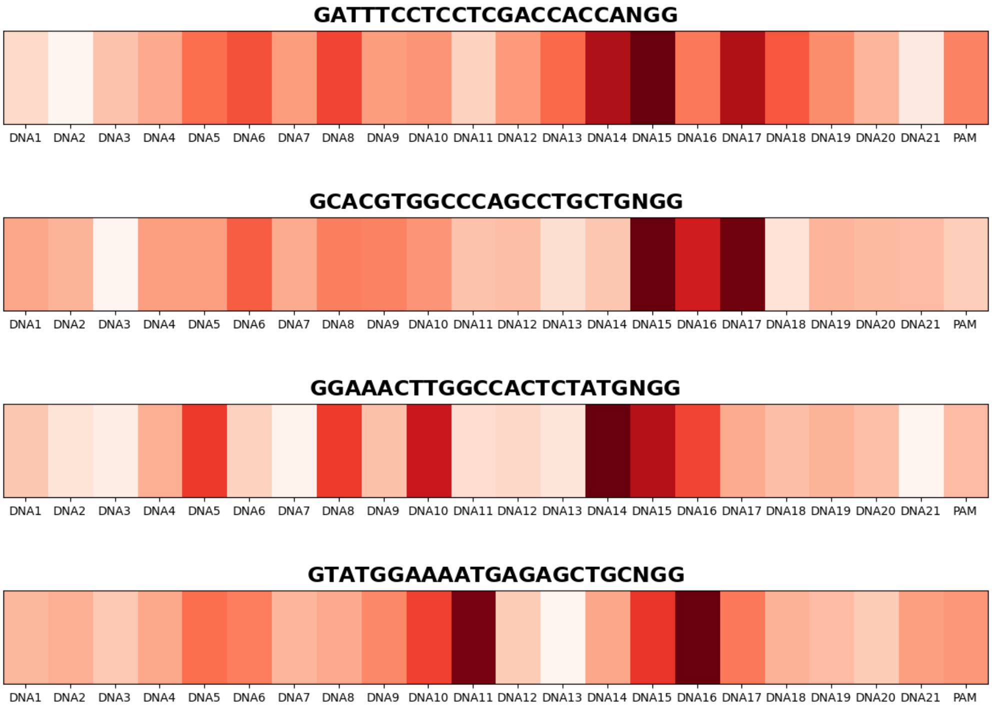

S4 Fig3. Integrated Gradients attribution heatmaps for the Lazzarotto *et al*. (2020) GUIDE-seq dataset from cross-validation fold 3rd of 14.

Each row displays a heatmap of attribution scores for a single sgRNA, with its sequence shown above. The columns correspond to the 3-mer token positions in the target DNA sequence, from the first token (DNA1) to the PAM site. Color intensity reflects the attribution score, where darker red indicates a stronger positive contribution to the model's prediction of an off-target event. The sgRNA sequence is displayed in bold if its maximum attribution score is located within the PAM-distal (positions 4–6) or PAM-proximal (positions 13‑17) hotspots. This analysis was performed using the DNABERT model trained on the corresponding data from this cross-validation fold.

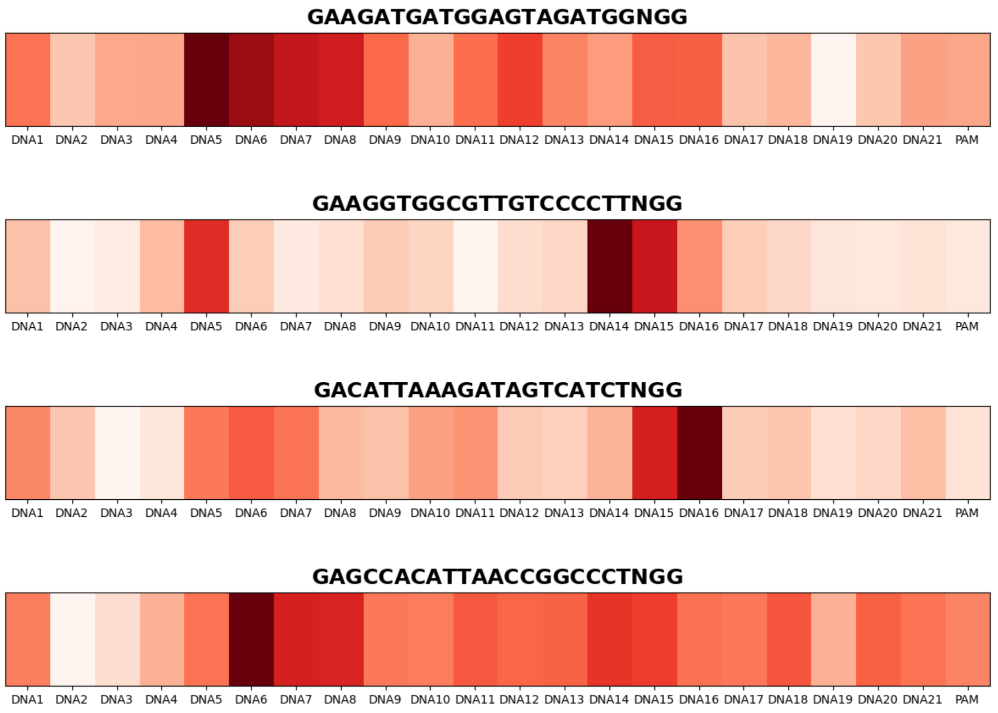

S4 Fig4. Integrated Gradients attribution heatmaps for the Lazzarotto *et al*. (2020) GUIDE-seq dataset from cross-validation fold 4th of 14.

Each row displays a heatmap of attribution scores for a single sgRNA, with its sequence shown above. The columns correspond to the 3-mer token positions in the target DNA sequence, from the first token (DNA1) to the PAM site. Color intensity reflects the attribution score, where darker red indicates a stronger positive contribution to the model's prediction of an off-target event. The sgRNA sequence is displayed in bold if its maximum attribution score is located within the PAM-distal (positions 4–6) or PAM-proximal (positions 13‑17) hotspots. This analysis was performed using the DNABERT model trained on the corresponding data from this cross-validation fold.

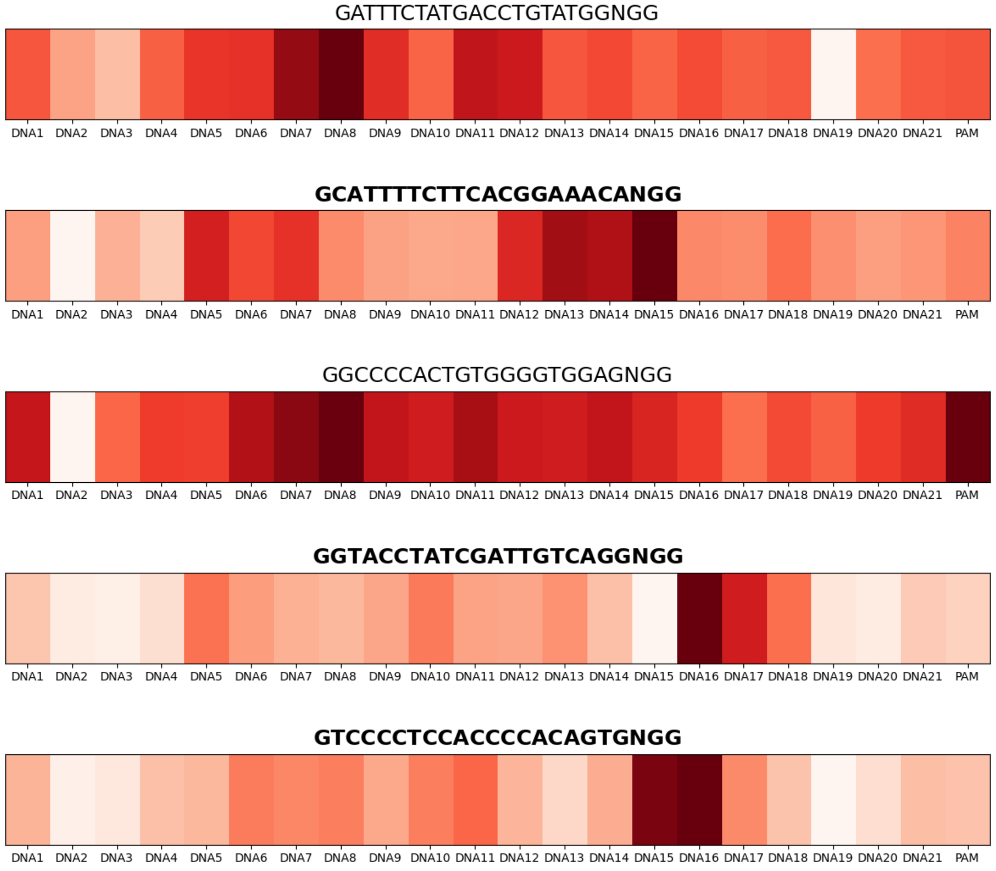

S4 Fig5. Integrated Gradients attribution heatmaps for the Lazzarotto *et al*. (2020) GUIDE-seq dataset from cross-validation fold 5th of 14.

Each row displays a heatmap of attribution scores for a single sgRNA, with its sequence shown above. The columns correspond to the 3-mer token positions in the target DNA sequence, from the first token (DNA1) to the PAM site. Color intensity reflects the attribution score, where darker red indicates a stronger positive contribution to the model's prediction of an off-target event. The sgRNA sequence is displayed in bold if its maximum attribution score is located within the PAM-distal (positions 4–6) or PAM-proximal (positions 13-17) hotspots. This analysis was performed using the DNABERT model trained on the corresponding data from this cross-validation fold.

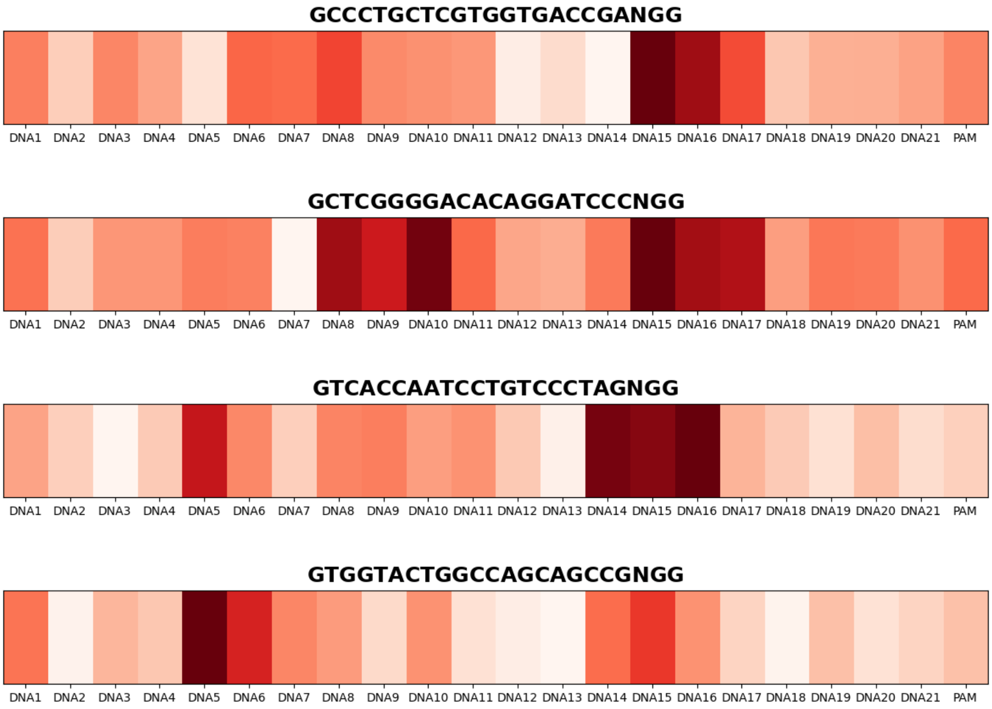

S4 Fig6. Integrated Gradients attribution heatmaps for the Lazzarotto *et al*. (2020) GUIDE-seq dataset from cross-validation fold 6th of 14.

Each row displays a heatmap of attribution scores for a single sgRNA, with its sequence shown above. The columns correspond to the 3-mer token positions in the target DNA sequence, from the first token (DNA1) to the PAM site. Color intensity reflects the attribution score, where darker red indicates a stronger positive contribution to the model's prediction of an off-target event. The sgRNA sequence is displayed in bold if its maximum attribution score is located within the PAM-distal (positions 4–6) or PAM-proximal (positions 13‑17) hotspots. This analysis was performed using the DNABERT model trained on the corresponding data from this cross-validation fold.

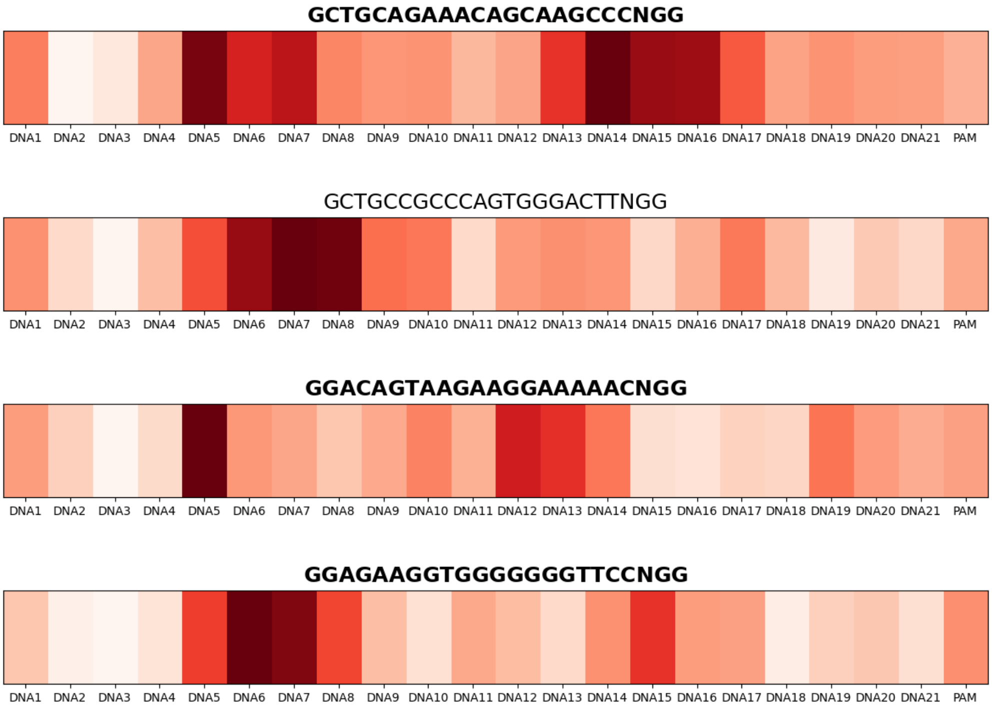

S4 Fig7. Integrated Gradients attribution heatmaps for the Lazzarotto *et al*. (2020) GUIDE-seq dataset from cross-validation fold 7th of 14.

Each row displays a heatmap of attribution scores for a single sgRNA, with its sequence shown above. The columns correspond to the 3-mer token positions in the target DNA sequence, from the first token (DNA1) to the PAM site. Color intensity reflects the attribution score, where darker red indicates a stronger positive contribution to the model's prediction of an off-target event. The sgRNA sequence is displayed in bold if its maximum attribution score is located within the PAM-distal (positions 4–6) or PAM-proximal (positions 13‑17) hotspots. This analysis was performed using the DNABERT model trained on the corresponding data from this cross-validation fold.

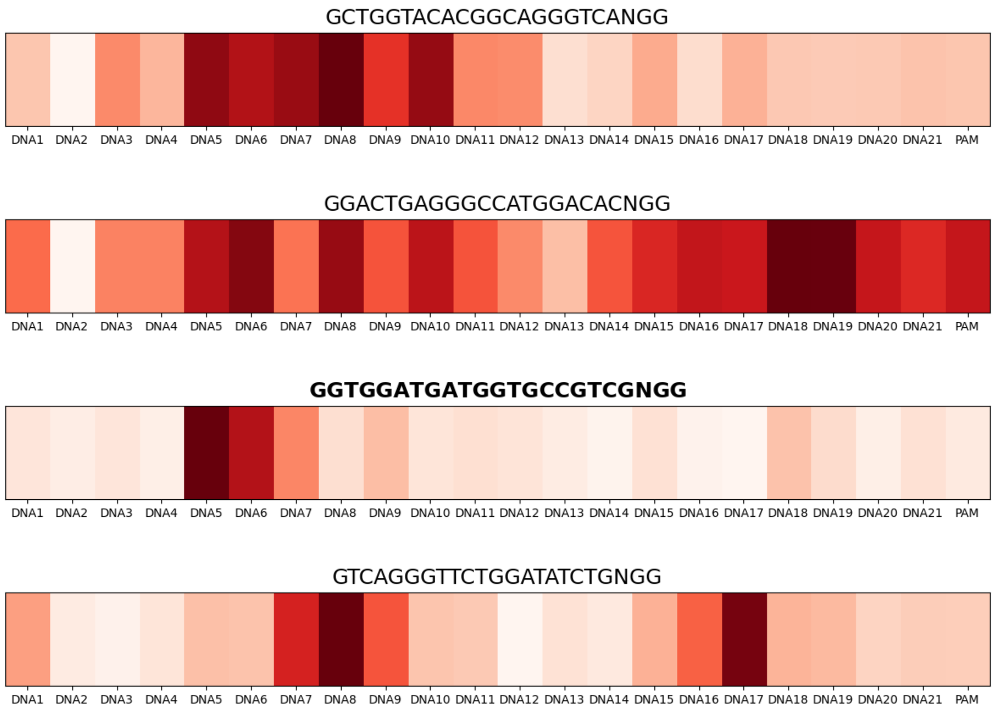

S4 Fig8. Integrated Gradients attribution heatmaps for the Lazzarotto *et al*. (2020) GUIDE-seq dataset from cross-validation fold 8th of 14.

Each row displays a heatmap of attribution scores for a single sgRNA, with its sequence shown above. The columns correspond to the 3-mer token positions in the target DNA sequence, from the first token (DNA1) to the PAM site. Color intensity reflects the attribution score, where darker red indicates a stronger positive contribution to the model's prediction of an off-target event. The sgRNA sequence is displayed in bold if its maximum attribution score is located within the PAM-distal (positions 4–6) or PAM-proximal (positions 13-17) hotspots. This analysis was performed using the DNABERT model trained on the corresponding data from this cross-validation fold.

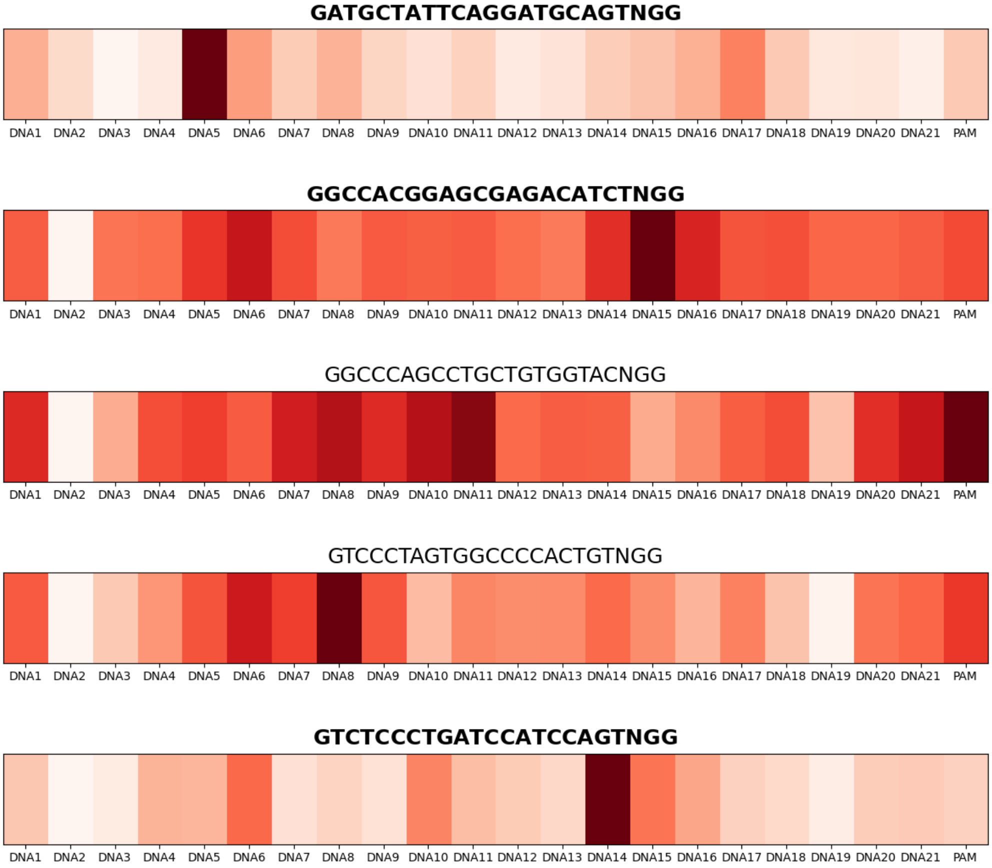

S4 Fig9. Integrated Gradients attribution heatmaps for the Lazzarotto *et al.* (2020) GUIDE-seq dataset from cross-validation fold 9th of 14.

Each row displays a heatmap of attribution scores for a single sgRNA, with its sequence shown above. The columns correspond to the 3-mer token positions in the target DNA sequence, from the first token (DNA1) to the PAM site. Color intensity reflects the attribution score, where darker red indicates a stronger positive contribution to the model's prediction of an off-target event. The sgRNA sequence is displayed in bold if its maximum attribution score is located within the PAM-distal (positions 4–6) or PAM-proximal (positions 13‐17) hotspots. This analysis was performed using the DNABERT model trained on the corresponding data from this cross-validation fold.

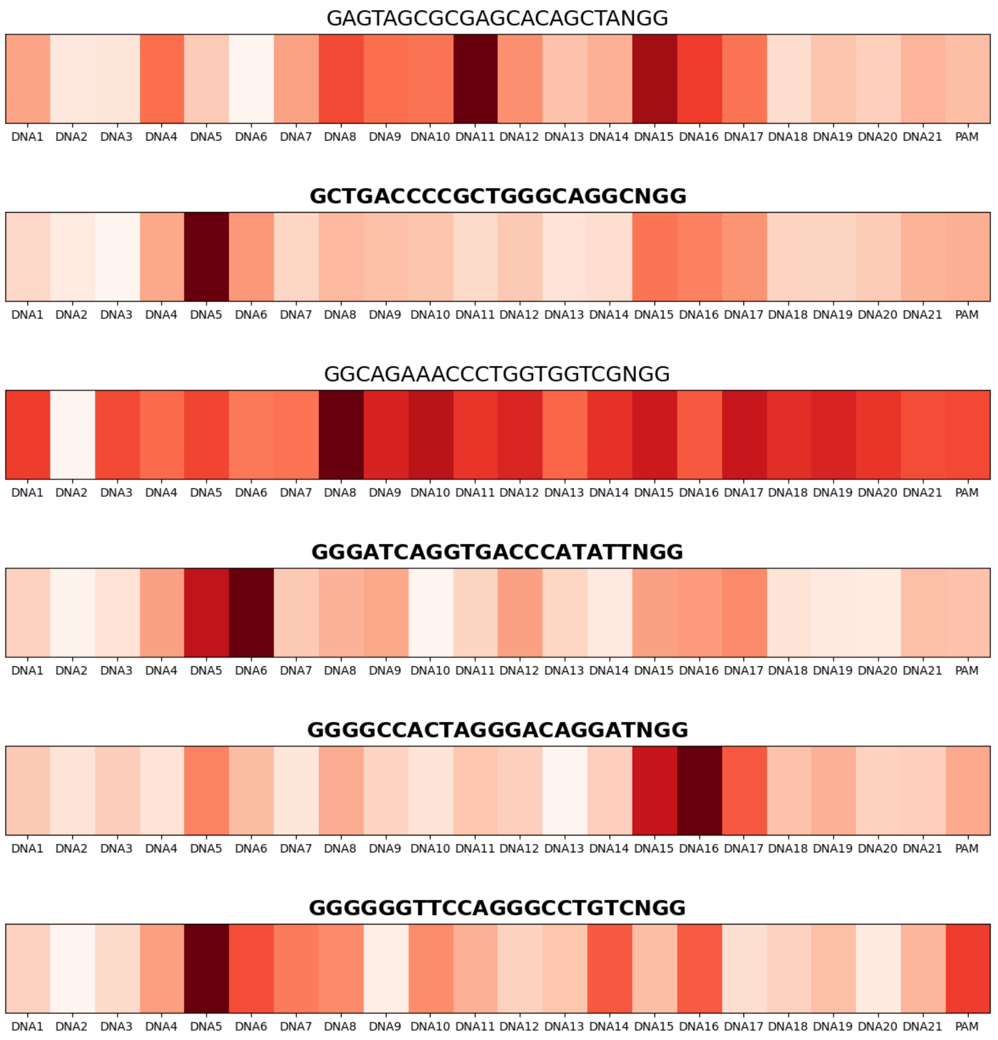

S4 Fig10. Integrated Gradients attribution heatmaps for the Lazzarotto *et al*. (2020) GUIDE-seq dataset from cross-validation fold 10th of 14.

Each row displays a heatmap of attribution scores for a single sgRNA, with its sequence shown above. The columns correspond to the 3-mer token positions in the target DNA sequence, from the first token (DNA1) to the PAM site. Color intensity reflects the attribution score, where darker red indicates a stronger positive contribution to the model's prediction of an off-target event. The sgRNA sequence is displayed in bold if its maximum attribution score is located within the PAM-distal (positions 4–6) or PAM-proximal (positions 13‑17) hotspots. This analysis was performed using the DNABERT model trained on the corresponding data from this cross-validation fold.

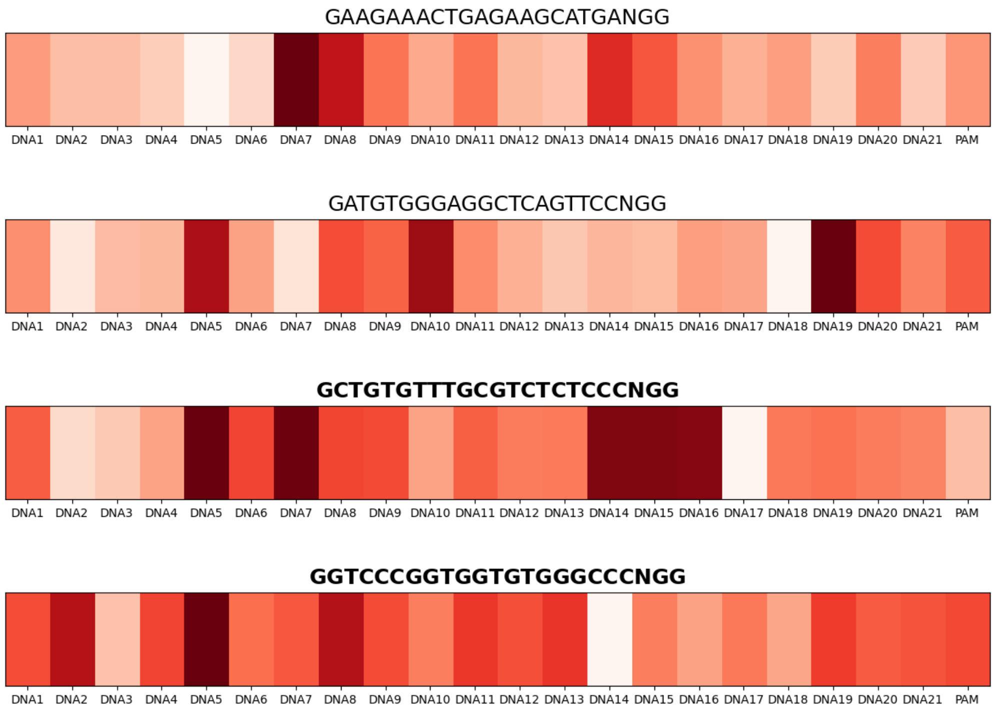

S4 Fig11. Integrated Gradients attribution heatmaps for the Lazzarotto *et al*. (2020) GUIDE-seq dataset from cross-validation fold 11th of 14.

Each row displays a heatmap of attribution scores for a single sgRNA, with its sequence shown above. The columns correspond to the 3-mer token positions in the target DNA sequence, from the first token (DNA1) to the PAM site. Color intensity reflects the attribution score, where darker red indicates a stronger positive contribution to the model's prediction of an off-target event. The sgRNA sequence is displayed in bold if its maximum attribution score is located within the PAM-distal (positions 4–6) or PAM-proximal (positions 13‑17) hotspots. This analysis was performed using the DNABERT model trained on the corresponding data from this cross-validation fold.

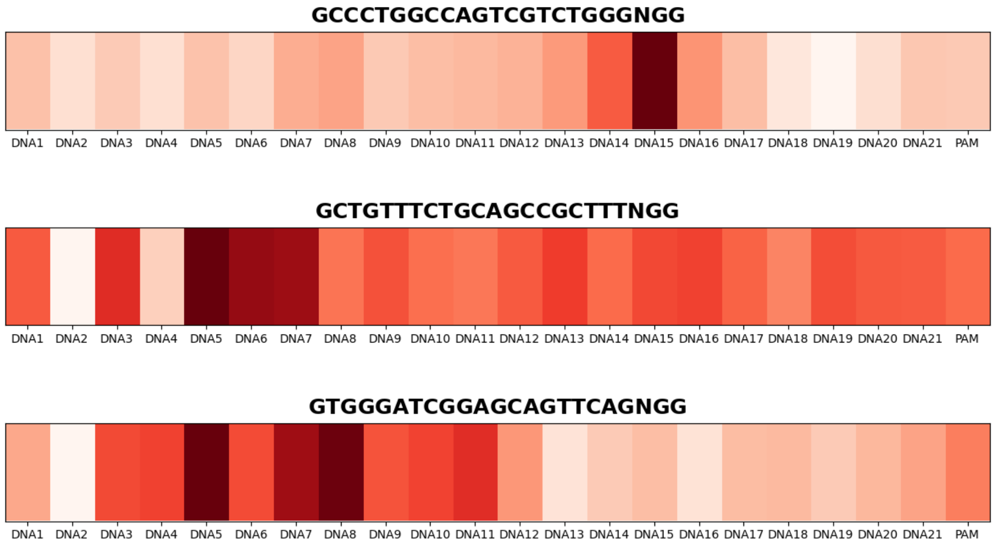

S4 Fig12. Integrated Gradients attribution heatmaps for the Lazzarotto *et al*. (2020) GUIDE-seq dataset from cross-validation fold 13th of 14.

Each row displays a heatmap of attribution scores for a single sgRNA, with its sequence shown above. The columns correspond to the 3-mer token positions in the target DNA sequence, from the first token (DNA1) to the PAM site. Color intensity reflects the attribution score, where darker red indicates a stronger positive contribution to the model's prediction of an off-target event. The sgRNA sequence is displayed in bold if its maximum attribution score is located within the PAM-distal (positions 4–6) or PAM-proximal (positions 13‐17) hotspots. This analysis was performed using the DNABERT model trained on the corresponding data from this cross-validation fold.

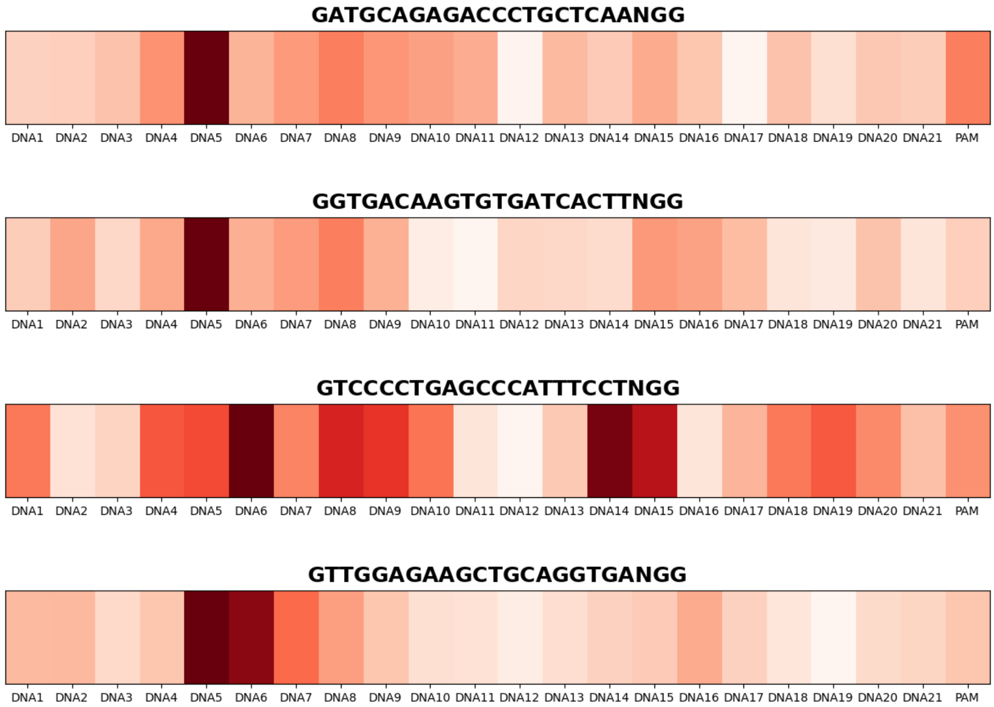

S4 Fig13. Integrated Gradients attribution heatmaps for the Lazzarotto *et al*. (2020) GUIDE-seq dataset from cross-validation fold 14th of 14.

Each row displays a heatmap of attribution scores for a single sgRNA, with its sequence shown above. The columns correspond to the 3-mer token positions in the target DNA sequence, from the first token (DNA1) to the PAM site. Color intensity reflects the attribution score, where darker red indicates a stronger positive contribution to the model's prediction of an off-target event. The sgRNA sequence is displayed in bold if its maximum attribution score is located within the PAM-distal (positions 4–6) or PAM-proximal (positions 13‑17) hotspots. This analysis was performed using the DNABERT model trained on the corresponding data from this cross-validation fold.

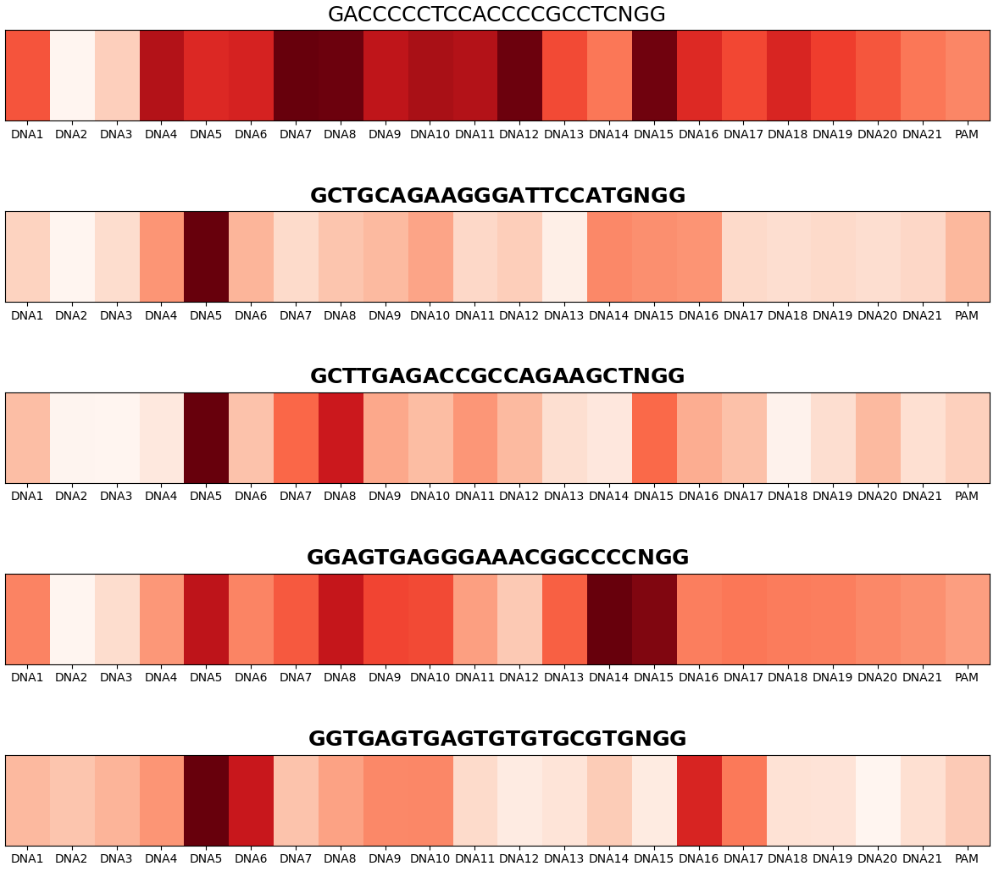

S4 Fig14. Integrated Gradients attribution heatmaps for the Chen *et al.* (2017) GUIDE-seq dataset.

Each row displays a heatmap of attribution scores for a single sgRNA, with its sequence shown above. The columns correspond to the 3-mer token positions in the target DNA sequence, from the first token (DNA1) to the PAM site. Color intensity reflects the attribution score, where darker red indicates a stronger positive contribution to the model's prediction of an off-target event. The sgRNA sequence is displayed in bold if its maximum attribution score is located within the PAM-distal (positions 4–6) or PAM-proximal (positions 13‑17) hotspots. This analysis was performed using the DNABERT model that was trained on the 12th fold of the Lazzarotto et al. (2020) GUIDE-seq cross-validation data.

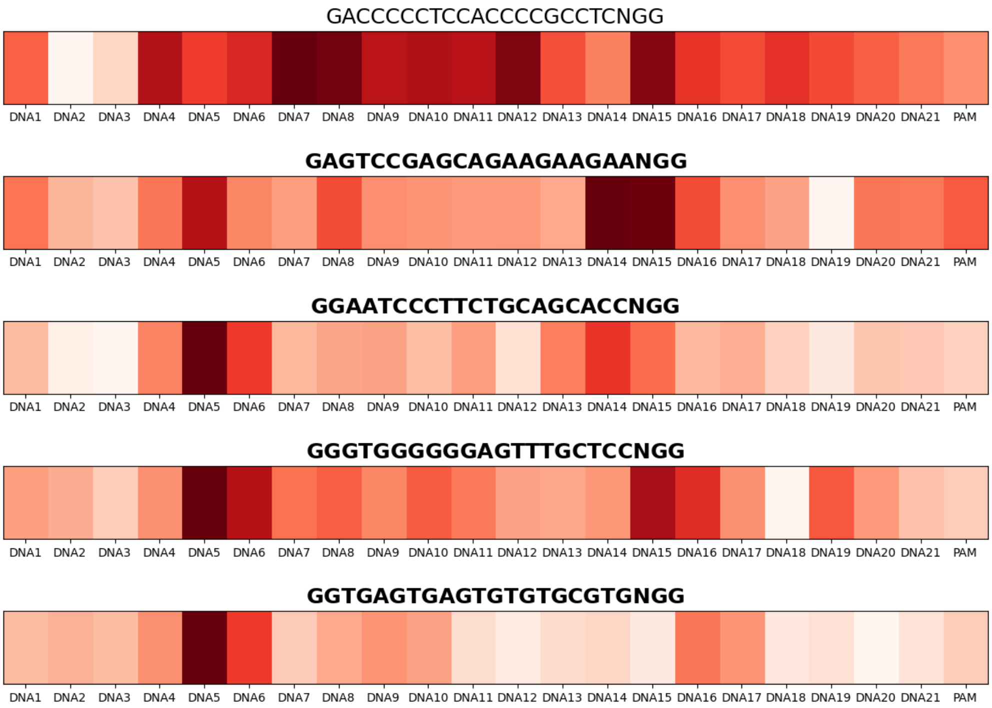

S4 Fig15. Integrated Gradients attribution heatmaps for the Tsai *et al.* (2015) GUIDE-seq U2OS dataset.

Each row displays a heatmap of attribution scores for a single sgRNA, with its sequence shown above. The columns correspond to the 3-mer token positions in the target DNA sequence, from the first token (DNA1) to the PAM site. Color intensity reflects the attribution score, where darker red indicates a stronger positive contribution to the model's prediction of an off-target event. The sgRNA sequence is displayed in bold if its maximum attribution score is located within the PAM-distal (positions 4–6) or PAM-proximal (positions 13-17) hotspots. This analysis was performed using the DNABERT model that was trained on the 12th fold of the Lazzarotto et al. (2020) GUIDE-seq cross-validation data.

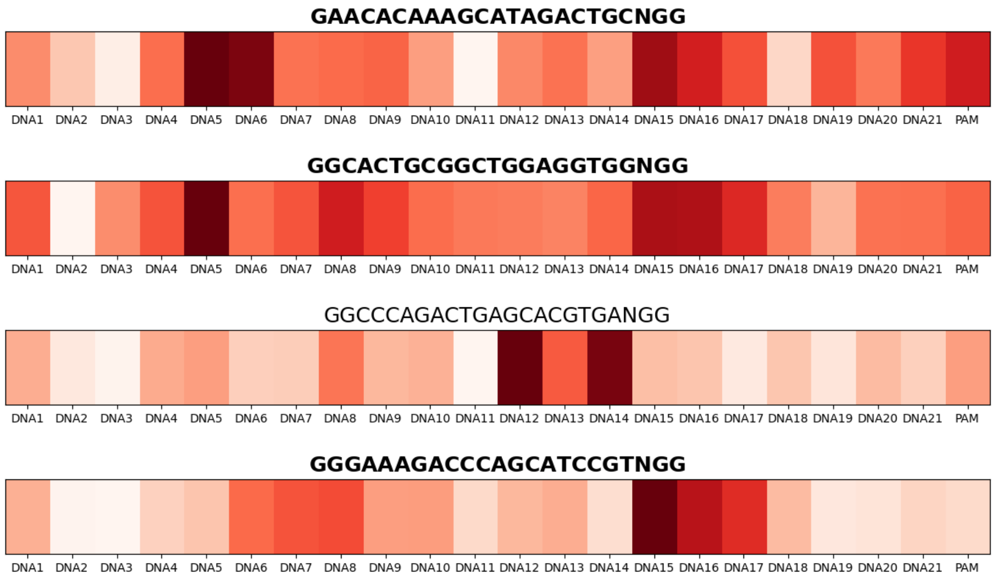

S4 Fig16. Integrated Gradients attribution heatmaps for the Tsai *et al.* (2015) GUIDE-seq HEK293 dataset.

Each row displays a heatmap of attribution scores for a single sgRNA, with its sequence shown above. The columns correspond to the 3-mer token positions in the target DNA sequence, from the first token (DNA1) to the PAM site. Color intensity reflects the attribution score, where darker red indicates a stronger positive contribution to the model's prediction of an off-target event. The sgRNA sequence is displayed in bold if its maximum attribution score is located within the PAM-distal (positions 4–6) or PAM-proximal (positions 13-17) hotspots. This analysis was performed using the DNABERT model that was trained on the 12th fold of the Lazzarotto et al. (2020) GUIDE-seq cross-validation data.
