# Supplementary figures and images for: Improved CRISPR/Cas9 off-target prediction with DNABERT and epigenetic features
Source: PLoS One. 2025 Nov 12;20(11):e0335863. doi: 10.1371/journal.pone.0335863 (PMC12611124; doi:10.1371/journal.pone.0335863)

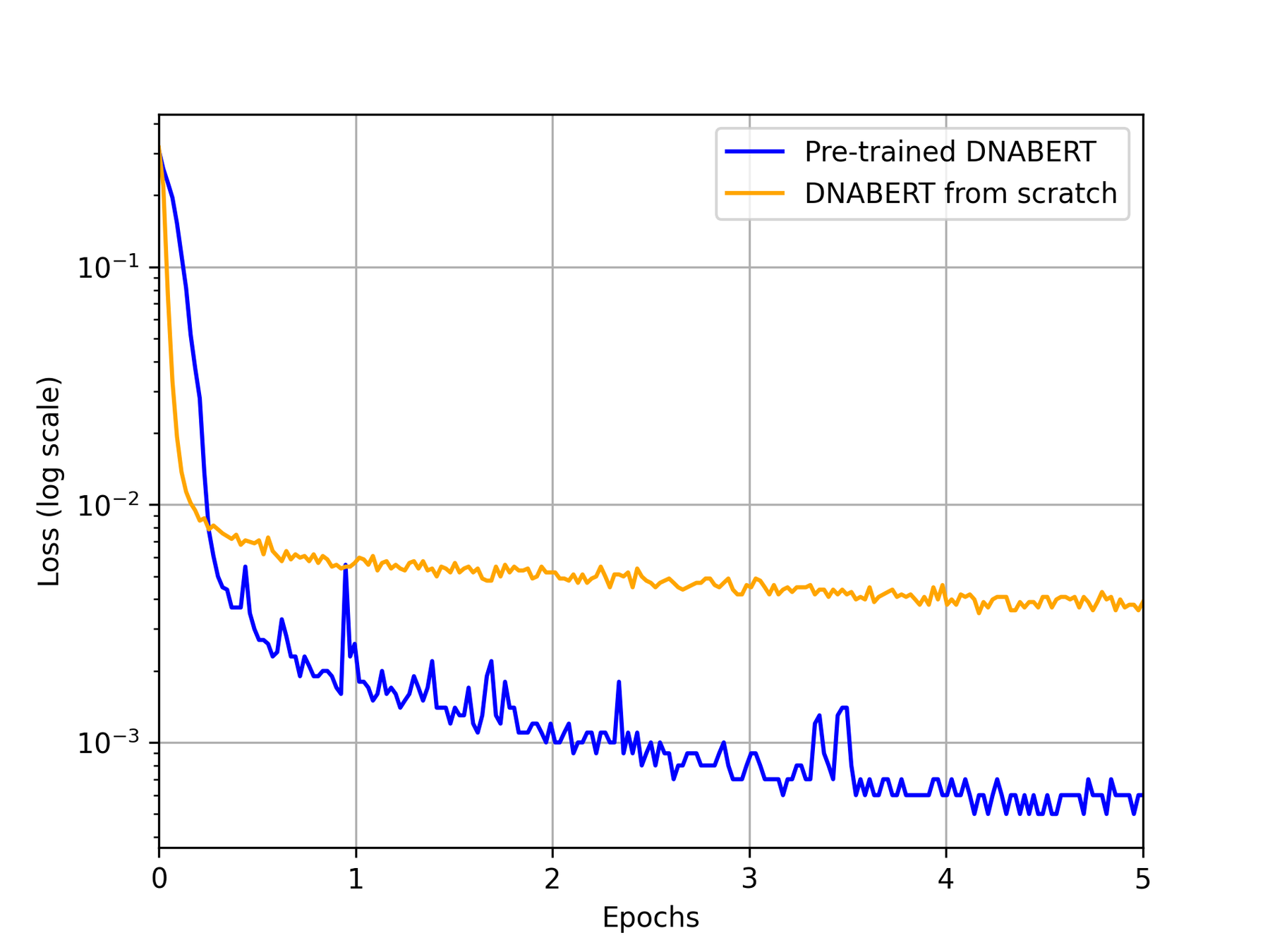

Supplement: S1 Fig — (TIFF) [file pone.0335863.s010.tiff]
